# The Gαi-GIV binding interface is a druggable protein-protein interaction

**DOI:** 10.1038/s41598-017-08829-7

**Published:** 2017-08-17

**Authors:** Vincent DiGiacomo, Alain Ibáñez de Opakua, Maria P. Papakonstantinou, Lien T. Nguyen, Nekane Merino, Juan B. Blanco-Canosa, Francisco J. Blanco, Mikel Garcia-Marcos

**Affiliations:** 1 Department of Biochemistry, Boston University School of Medicine, Boston, USA; 2 CIC-BioGune, Derio, Spain; 3 Department of Chemistry and Molecular Pharmacology, IRB Barcelona, Barcelona, Spain; 4 IKERBASQUE, Basque Foundation for Science, Bilbao, Spain

## Abstract

Heterotrimeric G proteins are usually activated by the guanine-nucleotide exchange factor (GEF) activity of GPCRs. However, some non-receptor proteins are also GEFs. GIV (a.k.a Girdin) was the first non-receptor protein for which the GEF activity was ascribed to a well-defined protein sequence that directly binds Gαi. GIV expression promotes metastasis and disruption of its binding to Gαi blunts the pro-metastatic behavior of cancer cells. Although this suggests that inhibition of the Gαi-GIV interaction is a promising therapeutic strategy, protein-protein interactions (PPIs) are considered poorly “druggable” targets requiring case-by-case validation. Here, we set out to investigate whether Gαi-GIV is a druggable PPI. We tested a collection of >1,000 compounds on the Gαi-GIV PPI by *in silico* ligand screening and separately by a chemical high-throughput screening (HTS) assay. Two hits, ATA and NF023, obtained in both screens were confirmed in secondary HTS and low-throughput assays. The binding site of NF023, identified by NMR spectroscopy and biochemical assays, overlaps with the Gαi-GIV interface. Importantly, NF023 did not disrupt Gαi-Gβγ binding, indicating its specificity toward Gαi-GIV. This work establishes the Gαi-GIV PPI as a druggable target and sets the conceptual and technical framework for the discovery of novel inhibitors of this PPI.

## Introduction

Trimeric G proteins regulate all kinds of physiological functions in humans and their dysregulation is the cause of many diseases^1–3^. They cycle between inactive (GDP-bound) and active (GTP-bound) states to control the flow of information from extracellular cues to intracellular effectors^3, 4^. In the classical model, resting Gα-GDP in complex with Gβγ is activated at the plasma membrane by G Protein-Coupled Receptors (GPCRs), which promote the exchange of GDP for GTP and dissociation of Gβγ^3, 4^. G protein inactivation is mediated by the intrinsic GTPase activity of Gα, which leads to the re-association of Gα-GDP with Gβγ. Considering the critical role of this signaling mechanism in human physiology, it is not surprising that >30% of marketed drugs target GPCRs^5^, which are the components of this signaling pathway most readily accessible to exogenous molecules. Nevertheless, other elements of this signal transduction mechanism have also gained interest as possible therapeutic targets. These include G proteins themselves as well as intracellular proteins that modulate their activity. For example, there are small molecules and natural products that target Gα or Gβγ subunits, and some of them have been validated in preclinical models of experimental therapeutics for pain, inflammation or heart failure^6–10^. Among G protein regulators, targeting members of the Regulators of G protein Signaling (RGS) family has been the most intensely explored^11–15^. RGS proteins are GTPase Activating Proteins (GAPs) that accelerate the rate of G protein deactivation and are involved in essentially all GPCR-G protein signaling. Although several small molecule inhibitors of RGS proteins have been reported to date, their efficacy in experimental therapeutics models remains to be investigated.

Targeting G proteins and/or their intracellular regulators is viewed as a promising alternative approach to targeting individual GPCRs for the treatment of diseases caused by the simultaneous dysregulation of multiple GPCR signaling pathways^9^. This is the case for cancer, in which upregulation of multiple GPCR-dependent pathways contributes to both oncogenesis and metastatic spread^1, 16^. This complexity is further increased by the fact that different arrays of GPCR-dependent pathways contribute to different stages of cancer progression and different cancer types^1, 17^. Thus, a strategy that targets common signaling hubs that drive GPCR-mediated oncogenic signaling may result in a more efficient therapy. In this regard, recent results with BIM-46174, a small molecule inhibitor of Gα subunits, are encouraging because they demonstrate that it can inhibit tumor cell growth and invasion in tissue culture conditions and animal models^18, 19^.

GIV (a.k.a. Girdin) is an intracellular regulator of trimeric G proteins and a promising target in cancer metastasis^20–32^. We originally showed that GIV expression is upregulated in highly invasive colon, breast, and pancreatic carcinoma cell lines^20, 31^ and others found that GIV depletion blunts metastasis in mouse models^23^. We also found that GIV expression correlated with invasion/metastasis in human colorectal tumors *in situ* and that it served as an independent prognostic marker for shortened survival^20^. Subsequent studies, including some with large cohorts of hundreds of patients, have independently confirmed the correlation between GIV expression and cancer progression towards invasive/metastatic stages and shortened survival in different cancer types like colon, breast, esophagus, liver, lung or gliomas^24–29, 32–34^. At the cellular level, GIV is required for efficient tumor cell migration, actin remodeling and activation of the oncogenic PI3K-Akt pathway^35, 36^; a set of features associated with prometastatic cell behavior^37, 38^.

From a mechanistic standpoint, GIV’s function of controlling the prometastatic behavior of tumor cells is determined by a novel and unique G protein activating motif^21, 22, 30^. Trimeric G proteins are activated upon nucleotide exchange (GDP → GTP), which is normally catalyzed by the Guanine nucleotide Exchange Factor (GEF) activity of a GPCR^3^. However, we found that GIV, a non-receptor protein, is also a GEF for α-subunits of the Gi subfamily (Gαi1, 2 and 3)^22, 30^ and that such GEF activity is associated with a well-defined motif of ~20–30 amino acids named the Gα-Binding and Activating (GBA) motif^21, 22, 30^. By using mutants that specifically disrupt the physical interaction between GIV’s GBA motif and Gαi proteins, we showed that GIV’s GEF activity is necessary and sufficient to drive tumor cell phenotypes associated with metastasis like increased cell migration, cytoskeletal remodeling and PI3K-Akt signaling hyperactivation. Another important observation is that GIV operates in the context of signaling triggered by receptors different from GPCRs that play an important role in cancer progression. More specifically, GIV’s GEF activity is required for enhancing tumor cell migration and PI3K-Akt signaling in response to receptor tyrosine kinases (RTKs)^39^ and integrins^40, 41^. Collectively, these findings indicate that disruption of the interaction between GIV and G proteins is a possible strategy against cancer metastasis.

Although disruption of the Gαi-GIV interface as a therapeutic approach might be an attractive idea from a conceptual standpoint, a hurdle for its actual implementation is that it would rely on inhibiting a protein-protein interaction (PPI). PPIs have been traditionally considered unsuitable targets for inhibition by small molecules, i.e., not “druggable”. On the other hand, evidence accumulated in the recent years indicates that some PPIs can actually be disrupted by small molecules^42, 43^ and some of these PPI inhibitors are currently under investigation for cancer therapy^44, 45^. Therefore, the druggability of a given PPI can be defined only by experimental demonstration, i.e., by directly testing whether a small molecule can disrupt the PPI. Here, we set out to investigate the druggability of the Gαi-GIV interaction. In doing so, we generated a pipeline of assays to screen and validate small molecule inhibitors of Gαi3-GIV coupling and characterized the mode of action of a compound that disrupts this interaction. By using a combination of computational structure modeling, NMR and biochemistry, we validate that this inhibitor works by competing with GIV for binding to Gαi3 without interfering with the binding of Gβγ, another Gαi3 interacting partner. These findings suggest that the Gαi-GIV PPI can be directly and specifically targeted by small molecules and sets the technical and conceptual framework for the discovery of novel inhibitors of this PPI.

## Results

### Computational prediction of duggability for the Gαi3:GIV interface

**Figure 1:**
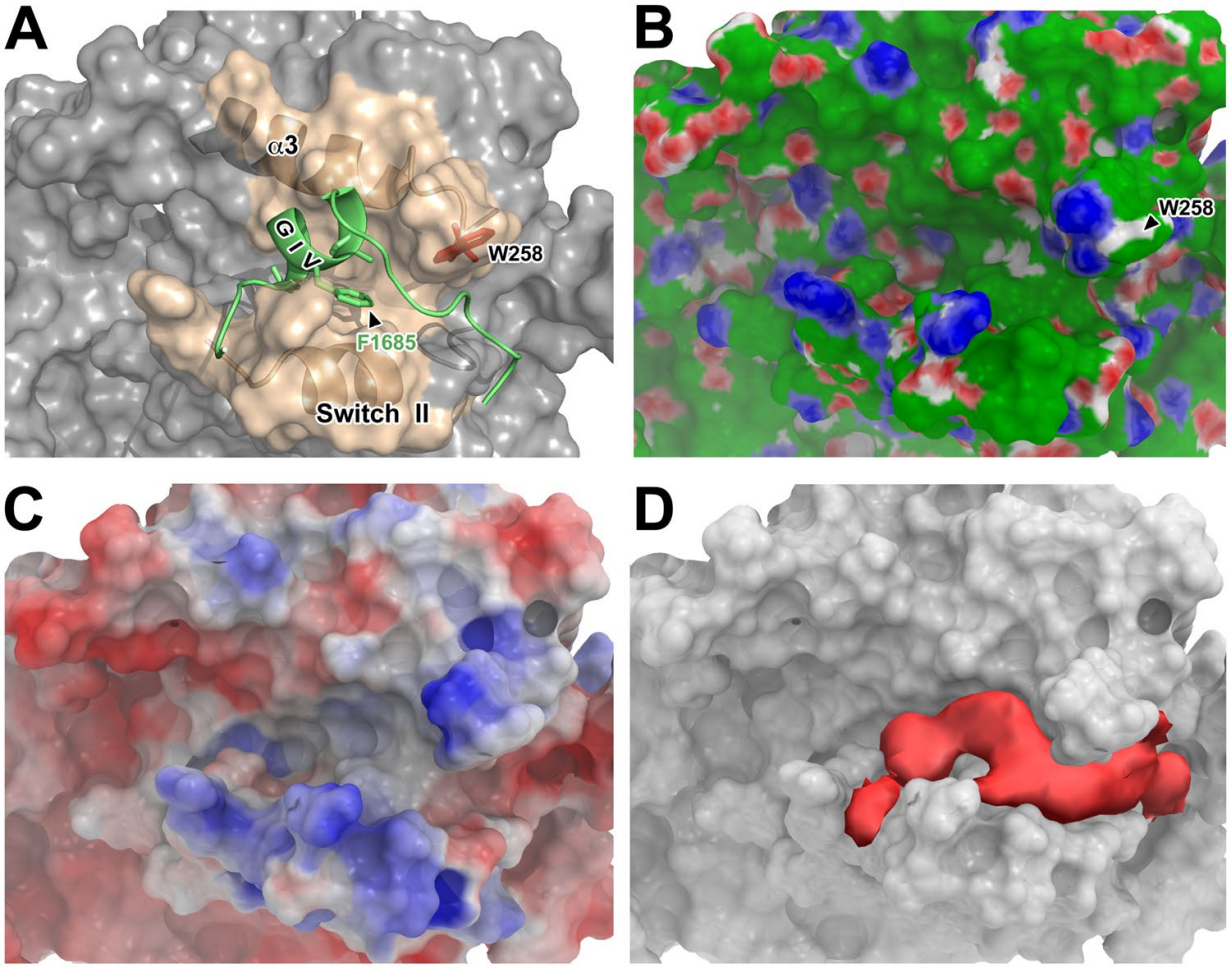
Computational prediction of a druggable site on the GIV binding region of Gαi3. (**A**) A model of GIV (residues 1678–1696, green ribbon) bound to Gαi3 (gray/beige surface) was built by homology modelling on the Gαi1:KB-752 crystal structure (PDB: 1Y3A) and *in silico* protein-protein docking as described in *Methods*. The interaction primarily involves an amphipathic α-helix of GIV engaging a hydrophobic cleft on Gαi3 (beige) formed by the α3 helix and the Switch II region. (**B**) Physicochemical properties of the GIV binding site on Gαi3. The same pose of the model as in (**A**) is shown displaying hydrophobic regions in green and hydrogen bond donors and acceptors in blue and red, respectively. GIV is not shown for the sake of clarity. (**C**) Electrostatic properties of GIV binding site on Gαi3. The same pose of the model as in (**A**) is shown displaying positively charged regions in blue, negatively charged in red and neutral in grey. GIV is not shown for the sake of clarity. **(D)** Predicted druggable pocket (red) on Gαi3 (gray). The pocket capable of accommodating small molecules was identified in the model depicted in (**A**) as described in *Methods*.

As a first step to assess the druggability of the Gαi-GIV interface, we analyzed its physicochemical properties computationally. For this we evaluated a previously generated structural model of GIV’s GBA motif bound to Gαi3^46^. Briefly, this model was constructed based on homology with the X-ray crystal structure of the GIV-like peptide KB-752 bound to Gαi1 to generate the GIV aa1678–1688 region followed by modeling *de novo* of the adjacent GIV aa1689–1696 region. The properties of the Gαi-GIV interaction predicted by this model have been extensively validated using site-directed mutagenesis and NMR spectroscopy^46^. Based on this model, the central portion of GIV’s GBA motif adopts a helical conformation that docks onto GDP-bound Gαi3. Previous biophysical and crystallographic studies have established that the Switch II region of Gαi-GDP is flexible and disordered in the absence of binding partners^47^. In contrast, GIV binding stabilizes a conformation of Switch II that creates a cleft framed by the α3 helix and Switch II region (Fig. 1A). The C-terminal non-helical segment of GIV’s GBA motif extends into a pocket framed by a surface-exposed tryptophan side chain (Gαi3 W258) located in the α3/β5 loop, a structural determinant previously shown to be important for GIV’s binding affinity and specificity^22^. Evaluation of the physicochemical properties of the GIV binding surface on Gαi3 revealed that it primarily consists of a hydrophobic cleft surrounded by polar groups (Fig. 1B,C). To assess the druggability of this interface, we used a “PocketFinder” algorithm, which identifies the regions of target proteins capable of accommodating small molecules^48^. This algorithm identified a pocket of ~435 Å^3^ that extends from beneath the W258 in the α3/β5 loop into the central region of the SwII/α3 cleft (Fig. 1D). Taken together, these computational analyses predict the existence of a pocket on Gαi3 that can accommodate small molecules and potentially disrupt GIV binding.

### Identification of a minimal Gαi-binding sequence in GIV

**Figure 2:**
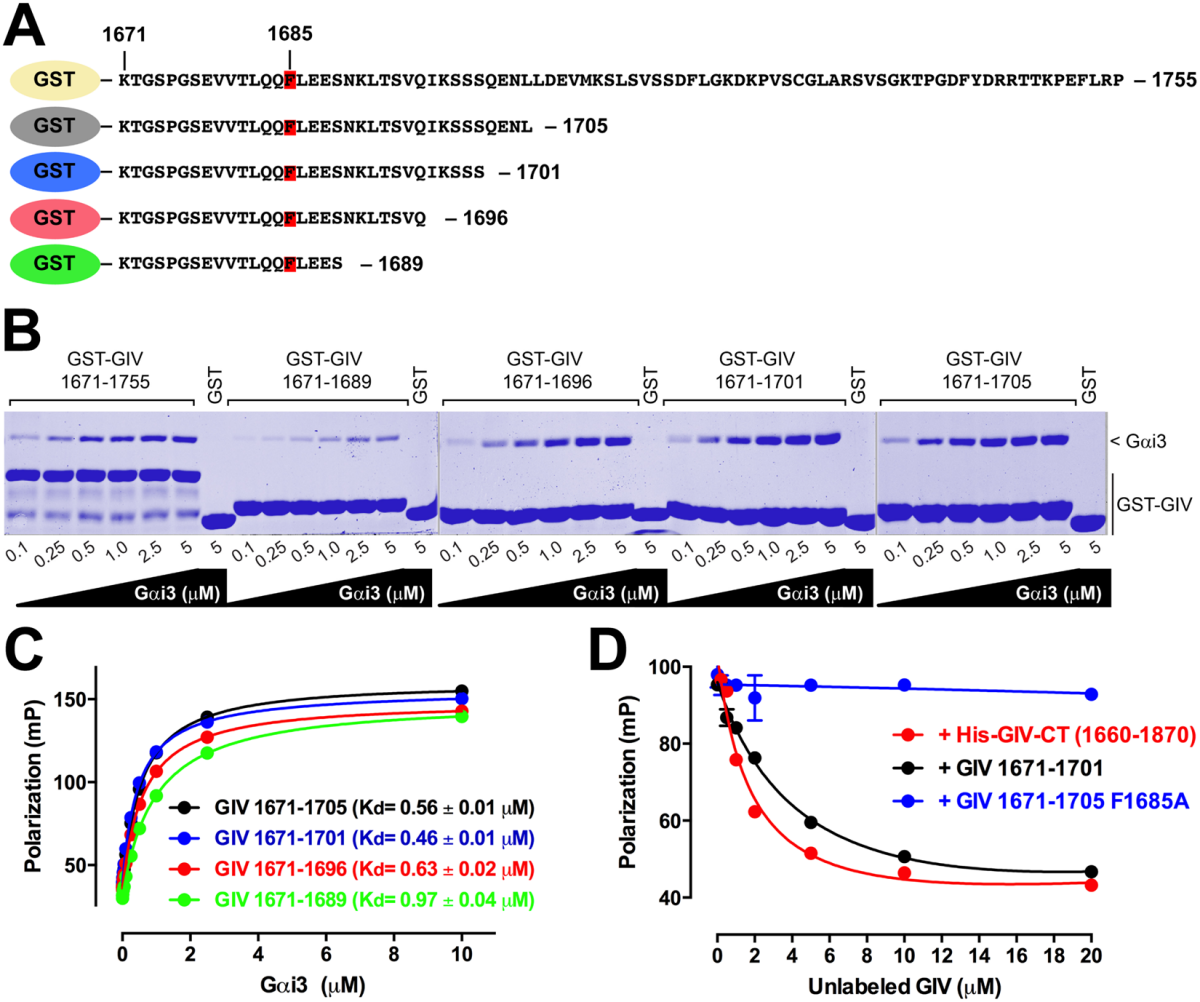
GIV 1671–1701 sequence is sufficient to bind Gαi3 with sub-µM affinity. (**A**) Scheme of GST-fused GIV sequences used to map the minimal Gαi3 binding region. F1685, a residue previously shown to be critical for Gαi3 binding, is shown in red. (**B**) Binding of purified Gαi3 to GST-GIV truncation constructs. Increasing concentrations of purified His-Gαi3 (0.1–5 µM) were incubated with different GST-GIV proteins and binding determined by pulldown assays as described in *Methods*. GST-GIV 1671–1689 showed diminished His-Gαi3 binding compared to the other GST-GIV proteins. No His-Gαi3 binding to the negative control GST was detected. One representative experiment out of three is shown. (**C**) Binding of purified Gαi3 to fluorescently-labeled GIV peptides. Increasing concentrations of purified His-Gαi3 (0.125–10 µM) were incubated with the indicated fluorescently-labeled GIV peptides (25 nM). Fluorescence polarization (FP) data were fit to a one-site binding model to calculate the equilibrium dissociation constant (K_d_) for each peptide. K_d_ results are expressed as mean ± S.E. of 3 independent experiments and the curves correspond to one representative experiment. (**D**) Competition of unlabeled GIV protein/peptides with fluorescently-labeled GIV 1671–1701 for binding to Gαi3. His-Gαi3 (1 µM) was incubated with fluorescently-labeled GIV 1671–1701 (25 nM) in the presence of increasing concentrations (0.5–20 µM) of His-GIV-CT (1660–1870, colored red) or unlabeled GIV 1671–1701 peptide (black). GIV 1671–1705 peptide containing the F1685A mutation was included as negative control (blue). Results are expressed as mean ± S.E. of 3 independent experiments (error bars not shown if smaller than the symbol). Images of the full gels presented in this figure are shown in Supplementary Information.

To empirically define if the Gαi-GIV PPI can be inhibited by small molecules, we set out to develop a high-throughput screening (HTS) assay. To develop this assay, we first performed experiments to identify the minimal GIV sequence that binds to Gαi3 with an affinity analogous to that of the native protein. Previous studies^22, 49, 50^ have established that GIV 1660–1870 (the C-terminal region containing its GBA motif) binds to Gαi3 with an equilibrium dissociation constant (K_d_) of ~0.3–0.5 µM and that this fragment recapitulates the biological functions of full-length GIV. It has also been shown that a shorter fragment of GIV (residues 1671–1755) fused to GST, binds to Gαi3 with the same affinity^51^. We created a battery of four new truncation constructs fused to GST in which the C-terminal sequence was progressively shortened from a length of 35 amino acids to only 19 (Fig. 2A). These constructs were purified and tested for binding to increasing amounts of purified His-Gαi3 in pulldown assays along with GST-GIV 1671–1755 for comparison (Fig. 2B). We found all the truncation constructs bound Gαi3 similar to GST-GIV 1671–1755, except for the shortest construct (GIV 1671–1689), which displayed diminished binding (Fig. 2B). These results suggest that GIV sequences spanning residues 1671–1696 or larger are sufficient to bind Gαi3 with an affinity similar to that of the native protein and that residues within the 1690–1696 region contribute significantly to the interaction.

In order to quantify the binding affinity of the truncated GIV segments for Gαi3, fluorescein-labeled peptides were synthesized and used in fluorescence polarization (FP) assays to calculate the K_d_ (Fig. 2C). Fluorescently-labeled GIV-derived peptides would be expected to yield an increase of FP upon binding to Gαi3 (~40 KDa). We found that the longest peptide (GIV 1671–1705) bound Gαi3 with K_d_ of ~0.5 µM, which is in agreement with the affinity of larger GIV fragments reported previously using pulldown assays^49, 51^. Binding of GIV 1671–1701 was indistinguishable from GIV 1671–1705, whereas the shorter GIV 1671–1696 peptide showed slightly diminished affinity (K_d_ ~0.65 µM) (Fig. 2C). Consistent with the results in pulldown experiments, GIV 1671–1689 peptide had a more marked decrease in affinity, showing ~2-fold weaker binding than the GIV 1671–1705 peptide (K_d_ ~1 µM) (Fig. 2C). These results indicate that GIV 1671–1701 (31 residues) is the shortest sequence tested that binds to Gαi3 with an affinity analogous to that of the native protein. To further confirm this and validate the FP assay, we performed competition experiments (Fig. 2D). For this purpose, a constant concentration of fluorescently-labeled GIV 1671–1701 peptide was incubated with Gαi3 in the presence of increasing concentrations of unlabeled GIV peptides/proteins. We found that unlabeled GIV 1671–1701, but not a control peptide containing an F1685A mutation (previously shown to disrupt GIV binding to Gαi3^21, 30^), efficiently decreased the FP signal in a dose-dependent manner. Consistent with this observation, we found that the F1685A mutation in GST-fused GIV 1671–1701 also impairs His-Gαi3 binding in pulldown assays (Supplementary Figure 1). These results validate the specificity of our FP assay for detecting GIV binding to Gαi3. In the same experiments we compared GIV 1671–1701 peptide with His-tagged GIV 1660–1870 (His-GIV-CT), a fragment that recapitulates the function of full-length GIV regarding Gαi3 protein^22, 49, 50^. Unlabeled GIV 1671–1701 peptide and His-GIV-CT competed with fluorescently-labeled GIV 1671–1701 with similar efficiency, indicating that both of them bind with comparable affinities for Gαi3 under the same experimental conditions. These results establish that GIV 1671–1701 is sufficient to recapitulate the binding properties of the native Gαi3-GIV interaction, thereby defining a minimal Gαi-binding sequence in GIV.

### Fluorescence polarization as a HTS-compatible assay to monitor the Gαi3-GIV interaction

**Figure 3:**
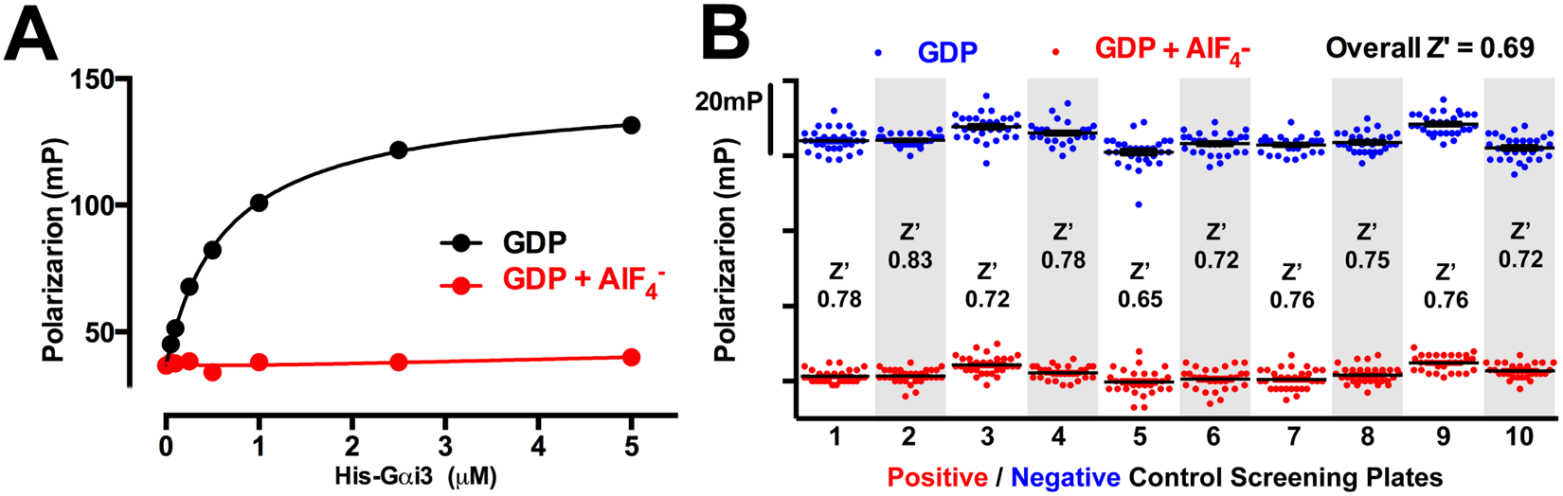
Assessment of the suitability of a Fluorescence Polarization (FP) assay for measuring Gαi3-GIV binding in a high-throughput format. (**A**) The GTP mimetic GDP+ AlF_4_^−^ blunts the Gαi3:GIV interaction as determined by FP. Binding of purified His-Gαi3 to the fluorescently-labeled GIV 1671–1701 peptide was determined as in Fig. 2C in the presence of GDP (black) or GDP+ AlF_4_^−^ (red). Increasing concentrations of purified His-Gαi3 (0.125–10 µM) were incubated with the peptide (25 nM). Fluorescence polarization (FP) data were fit to a one-site binding model. One representative experiment out of three is shown. (**B**) Scatter plot of positive and negative control data points and Z’ determination for the Gαi3:GIV 1671–1701 peptide binding FP assay. FP measurements were carried out in a 384-well format in ten independent plates for an equal number of wells with assay buffer containing GDP alone (blue, negative controls) or GDP+ AlF_4_^−^ (red, positive controls). Z’ for each plate was calculated as described in *Methods* and the overall Z’ was determined by pooling all data across the ten plates.

To experimentally assess the druggability of the Gαi3-GIV interface it is necessary to test hundreds to thousands of compounds in an assay format compatible with HTS. The FP assay described above has the potential to serve this purpose because it can be used in a “mix-and-read” miniaturized format. To directly evaluate the HTS compatibility of this assay we determined the Z’ (a.k.a Z-factor), a parameter that determines the robustness of an assay by taking into account the dynamic range of the signals between positive and negative control signals and the standard deviation of the measurements. We reasoned that addition of AlF_4_^−^ to the reactions would serve as a positive control for inhibition because previous work has demonstrated that GIV binds to Gαi when loaded with GDP but not when loaded with GDP·AlF_4_^−^ (which mimics the GTP-bound transition state) or with GTPγS (a non-hydrolysable GTP analog)^22, 30^. As expected, FP measurements carried out in the presence of GDP revealed robust binding of GIV to Gαi3 whereas binding was essentially abolished in the presence of GDP+ AlF_4_^−^ (Fig. 3A). DMSO concentrations up to 2.5% did not affect GIV binding to Gαi3 in this assay (Supplementary Figure 2). Next, we determined the Z’ value in 10 independent sets (plates) by analyzing the FP values in the presence or absence of AlF_4_^−^ in a 384-well plate format. The results revealed Z’ values ranging from 0.65 to 0.83 in individual plates and an overall Z’ of 0.69 (Fig. 3B). Because Z’ values >0.5 are typically considered robust enough for HTS, these results validate that our FP assay is compatible with HTS.

### Identification of Gαi3-GIV inhibitor hits by FP-HTS and *in silico* screening

**Figure 4:**
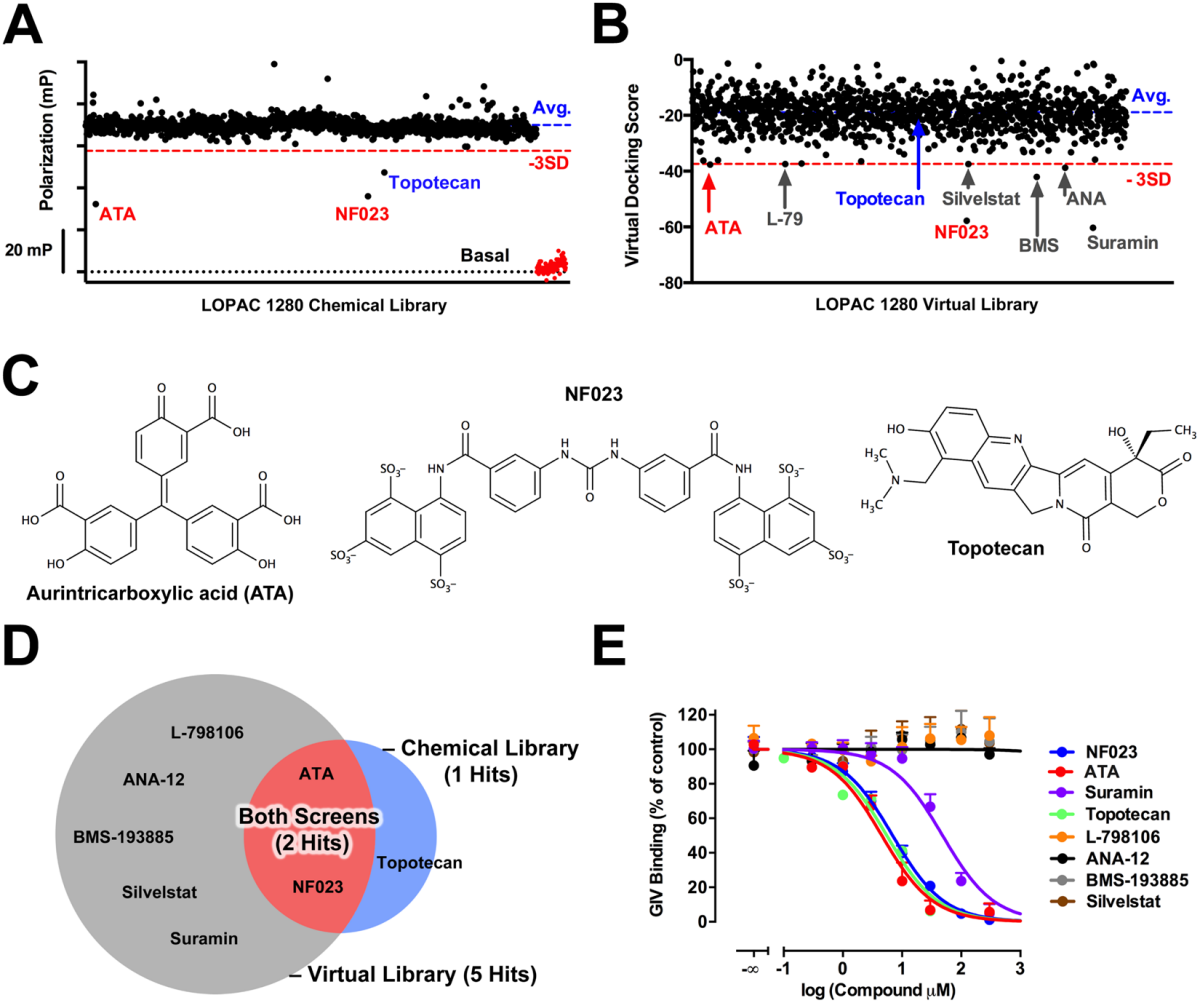
Identification of potential inhibitors of the Gαi3-GIV interaction by FP-HTS or *in silico* screening. (**A,B**) The LOPAC library of 1,280 small bioactive molecules was screened using an FP assay (**A**) or by virtual ligand screening (**B**) as described in *Methods*. Results are presented as scatter plots in which black dots represent the result for each individual compound (polarization or docking scores in the Y-axis). Red dots in (**A**) correspond to the positive controls for inhibition (GDP+ AlF_4_^−^) and the black dotted line the basal polarization observed for the free fluorescent peptides in the absence of Gαi3. The red dashed line is the value corresponding to the average minus 3 standard deviations (−3SD), which was established as the cut-off value to consider compounds as hits. Compound names are color coded as follows: red = hits identified in both screens, blue = hits identified only in the FP screen, and grey = hits identified only in the virtual ligand screen. ANA = ANA-12; L-79 = L-798106, BMS = BMS-193885. **(C)** Chemical structures of ATA, NF023 and Topotecan, the 3 hits identified in the FP-based screen shown in (**A**). (**D**) Venn diagram depicting the overlap of hits obtained by FP-HTS and *in silico* screening of the LOPAC library. (**E**) Dose-inhibition curves of hits. All hit compounds (total = 8) identified as potential inhibitors of the Gαi3:GIV interaction by FP and/or virtual docking were tested at different concentrations (0.1–300 µM) in FP assays. FP data was normalized relative to maximal binding in the absence of compounds and fitted to a one-site sigmoidal inhibition curve as described in *Methods*. Results are expressed as mean ± S.E. of 5 independent experiments.

To directly assess the druggability of the Gαi3-GIV PPI, we screened the Library of Pharmacologically Active Compounds (LOPAC), a collection of 1,280 diverse bioactive molecules that are commercially available. This library was screened in parallel using two independent approaches: one was our HTS-compatible FP assay and the other one was based on small molecule docking *in silico* using our Gαi3-GIV structural model. The goal of using this two-pronged approach was to assess the value of our structural model to predict the binding of small molecules. The FP-based HTS resulted in 3 hits as defined by molecules that decrease the FP signal below 3 standard deviations (3 SD) of the average of negative controls (Fig. 4A). For the *in silico* screen, we used a Monte Carlo-based computational approach to dock the individual molecules of the LOPAC onto the GIV interacting region on Gαi3 defined by our structural model (Fig. 1). Each screened molecule received a docking score reflecting compound binding probability (lower scores correspond to more favorable docking). Seven compounds with docking scores below the average minus 3 SD were considered hits (Fig. 4B). Two compounds, ATA and NF023, were overlapping hits present in the two screens, resulting in a total of 8 unique hits from the two approaches (Fig. 4C). All 8 unique hits were tested in dose-inhibition curves (0–100 µM) by FP. The 2 hits overlapping in the two screens (ATA and NF023) showed a dose-dependent inhibition with IC_50_ of ~5 µM (Fig. 4D). The third hit from the FP HTS (Topotecan) displayed a similar IC_50_. Among the five remaining hits that were identified exclusively in the *in silico* screen, only one (suramin) showed an inhibition in the range of concentrations tested. The IC_50_ for suramin was approximately one order of magnitude higher than for the other inhibitors (IC_50_ ~50 µM), which probably explains why it was not identified as a hit in the FP assay performed at a 10 µM compound concentration. The fact that suramin has a docking score similar to NF023 (Fig. 4B) but it shows weaker inhibition in biochemical experiments (Fig. 4D) probably reflects a limitation of the *in silico* approach. Interestingly, NF023 is a derivative of suramin and it has been previously reported that both molecules can directly bind and inhibit Gα proteins, NF023 being a more potent inhibitor of Gαi than suramin^52^. These previous observations are consistent with the identification of NF023 and suramin as inhibitors of the Gαi3-GIV interaction and with their relative potency. To further characterize the activity of suramin-derivatives as inhibitors of the Gαi3-GIV interaction, we investigated the effect of NF449. NF449 is a suramin analog previously reported to inhibit Gαs but not Gαi activity^53^, which suggests that it might bind to the former but not the latter. However, we found that NF449 inhibits Gαi3-GIV binding with a potency similar to that of NF023 (Supplementary Figure 3). The most likely explanation to reconcile our findings with the previously reported selectivity of NF449 toward Gαs is that the compound binds to both Gαs and Gαi but can only inhibit the activity of Gαs. In fact, the previously reported selectivity of NF449 for Gαs vs Gαi was based only on G protein activity and direct binding of NF449 was not investigated^53^. Collectively, these results suggest that the Gαi3-GIV PPI can be disrupted by small molecules, including compounds previously reported to inhibit Gα proteins, and that the structural model of this PPI is sufficiently accurate to predict the binding of small molecules.

### Development and implementation of a secondary assay to confirm screen hits

To validate that the identified hits are *bona fide* inhibitors of the Gαi-GIV PPI and not just compounds that interfere non-specifically with the FP assay, we developed a secondary assay that detects GIV binding to Gαi3 using a different principle. More specifically, we used an AlphaScreen®-based assay. Briefly, purified His-GIV-CT 1660–1870 was coupled to donor beads and purified GST-Gαi3 was coupled to acceptor beads. Upon binding of GIV to Gαi3, donor and acceptor beads come in close proximity, which results in a chemiluminescent signal. This system is well suited to identify molecules that may interfere non-specifically with the FP readout because it operates at different wavelengths.

**Figure 5:**
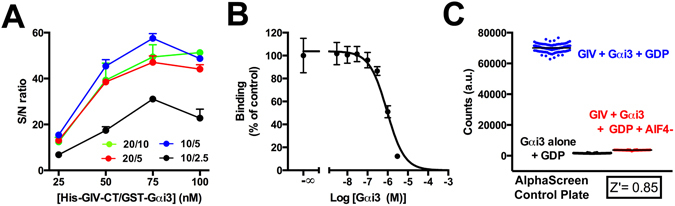
Validation of an AlphaScreen assay to measure Gαi3-GIV binding and assessment of its suitability for high-throughput screening. (**A**) Optimization of reagent concentrations to obtain maximal signal-to-noise ratio (S/N) in AlphaScreen® assays. The indicated concentrations of His-GIV-CT (1660–1870) and GST-Gαi3 (equimolar; represented in the X-axis) were incubated in the presence of the indicated concentrations (µg/ml) of AlphaScreen donor and acceptor beads. S/N ratios (represented in the Y-axis) were determined dividing the AlphaScreen® signal in the presence of both GIV and Gαi3 by the signal detected in the presence of Gαi3 alone. Results are expressed as mean ± S.E. of 3 independent experiments. (**B**) Inhibition of the Gαi3/GIV binding signal in AlphaScreen assays by untagged Gαi3. 75 nM proteins (His-GIV-CT and GST-Gαi3 protein), 10 μg/mL of donor beads and 5 μg/mL of acceptor beads were incubated in the presence of the indicated concentrations of unlabeled Gαi3. AlphaScreen signals were normalized to the value in the absence of untagged Gαi3 and fitted to a one-site sigmoidal inhibition curve as described in *Methods*. Results are expressed as mean ± S.E. of 3 independent experiments. (**C**) Scatter plot of positive and negative control data points and Z’ determination for the Gαi3-GIV binding AlphaScreen assay. Measurements were carried out in a 384-well format with 75 nM proteins (His-GIV-CT and GST-Gαi3 protein), 10 μg/mL of donor beads and 5 μg/mL of acceptor beads in buffer containing GDP alone (blue, negative controls) or GDP+ AlF_4_^−^ (red, positive controls). An additional condition in the presence of GDP but omitting His-GIV-CT was also included (black dots). Z’ was calculated from the positive and negative control data points as described in *Methods*.

First, we titrated the amounts of protein and beads to obtain the best signal to noise (S/N) ratio while minimizing the use of costly AlphaScreen® reagents. Titration of the protein concentrations is critical because it is known that excess of protein relative to the binding sites on the beads results in decreased signal (i.e., bell-shaped concentration-dependence curves). We determined the S/N ratios at 4 different concentrations of protein (25, 50, 75, 100 nM each for His-GIV-CT and GST-Gαi3) and 4 different ratios of donor/acceptor beads (Fig. 5A). S/N ratios were calculated by dividing the AlphaScreen® signal in the presence of both GIV and Gαi3 by the signal when only Gαi3 was present. We found that the maximal S/N ratio was obtained at a protein concentration of 75 nM and that the lowest amounts of beads that still yielded maximal S/N ratios were 10 and 5 µg/ml for donor and acceptor beads, respectively (Fig. 5A).

Using the conditions described above, next we validated that the AlphaScreen® signal specifically detects binding between GIV and Gαi3. First, we performed competition experiments with untagged Gαi3. Binding of untagged Gαi3 free in solution to bead-bound His-GIV-CT would displace the binding of bead-bound GST-Gαi3, which would be expected to decrease the AlphaScreen® signal. We found that this is the case, as increasing concentrations of untagged Gαi3 efficiently decreased the AlphaScreen® signal in a dose-dependent manner (Fig. 5B). The IC_50_ was ~1 µM, which is in good agreement with the previously estimated affinity of the Gαi3-GIV interaction. As a second approach to validate the specificity of the AlphaScreen® assay, we used AlF_4_^−^ as a positive control for inhibition. As expected, the AlphaScreen® signal was abolished in the presence of GDP+ AlF_4_^−^ vs GDP alone (Fig. 5C), reaching values almost identical to those of control reactions lacking one of the two binding partners (i.e., GST-Gαi3 alone). Using the GDP+ AlF_4_^−^ and GDP alone conditions as positive and negative controls, we determined that the assay has a Z’ of 0.85 (Fig. 5C), making it suitable for HTS. DMSO concentrations up to 5% did not affect GIV binding to Gαi3 in this assay (Supplementary Figure 4).

**Figure 6:**
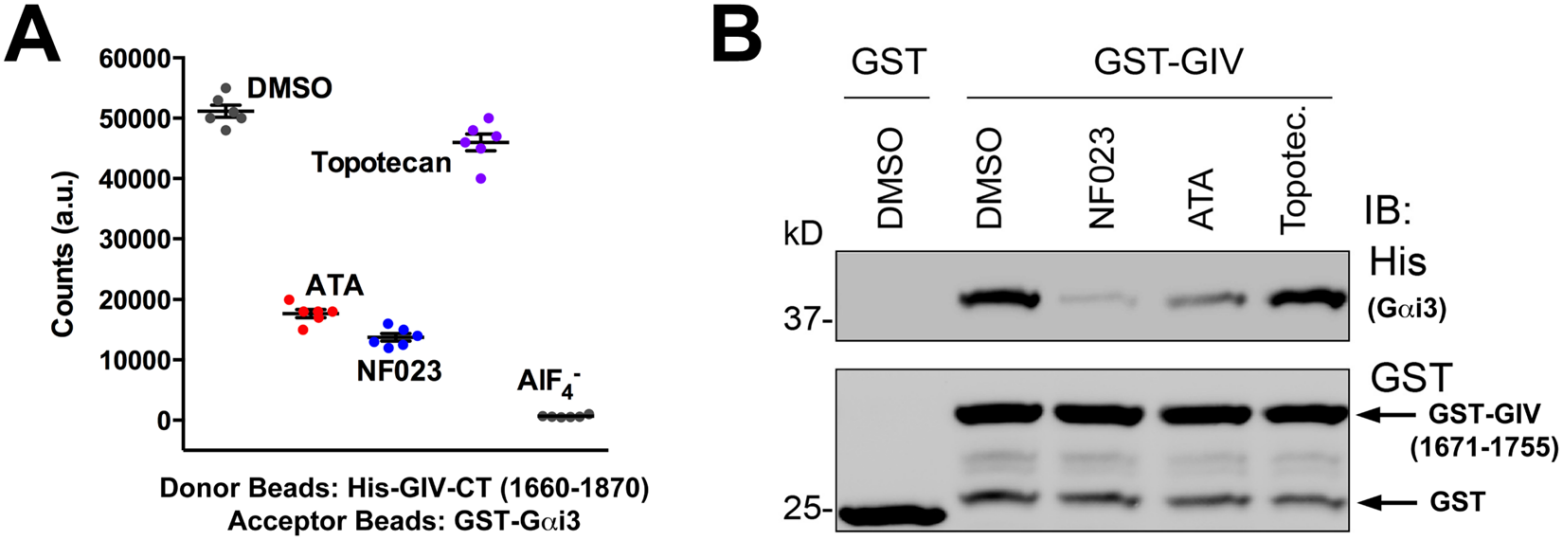
Validation of Gαi3-GIV inhibitor hits by independent assays. (**A**) ATA and NF023, but not topotecan inhibit Gαi3-GIV binding as determined by the AlphaScreen assay described in Fig. 5. 75 nM proteins (His-GIV-CT and GST-Gαi3 protein), 10 μg/mL of donor beads and 5 μg/mL of acceptor beads were incubated in the presence of the indicated compounds (10 µM) or an equivalent volume of DMSO. Conditions with AlF_4_^−^ were also included as positive controls. Data points correspond to six technical replicates of one representative experiment out of three. (**B**) ATA and NF023, but not topotecan inhibit Gαi3-GIV binding as determined by pulldown assays. GST-GIV 1671–1755 was immobilized on glutathione-agarose beads and incubated with His-Gαi3 in the presence of 100 µM of the indicated compounds. Resin-bound proteins were separated by SDS-PAGE and stained with Ponceau S or immunoblotted as indicated. One representative experiment out of three is shown. Imagens of the full membrane strips probed with antibody are shown in Supplementary Information.

Finally, we implemented this assay to validate the inhibitors previously shown (Fig. 4) block the Gαi3-GIV interaction in FP assays. We found that ATA and NF023 efficiently inhibited Gαi3-GIV binding as determined by AlphaScreen® (Fig. 6A). We also found that suramin is a less potent inhibitor than NF023 in the same AlphaScreen® assay (Supplementary Figure 5), which is in good agreement with the rank of potency observed in FP assays (Fig. 4). On the other hand, Topotecan did not inhibit Gαi3-GIV binding in AlphaScreen® assays (Fig. 6A), indicating that Topotecan is a false positive that probably interferes non-specifically with the FP assay. Indeed, we found that Topotecan is fluorescent at the wavelengths used in our FP assay (*data not shown*). Pulldown experiments with GST-GIV 1671–1755 and His-Gαi3 further confirmed that ATA and NF023 are *bona fide* Gαi3-GIV inhibitors whereas Topotecan is a false positive hit (Fig. 6B). Importantly, our *in silico* screen (Fig. 4B) predicted a very poor docking score for Topotecan, which in light of the results of our confirmatory assays supports the predictive power of our structural model. Together with data presented in preceding sections, these results validate a pipeline of assays to identify and validate inhibitors of the Gαi-GIV PPI in a high-throughput format.

### Mapping of NF023 binding site on Gαi3 by computational and NMR approaches

**Figure 7:**
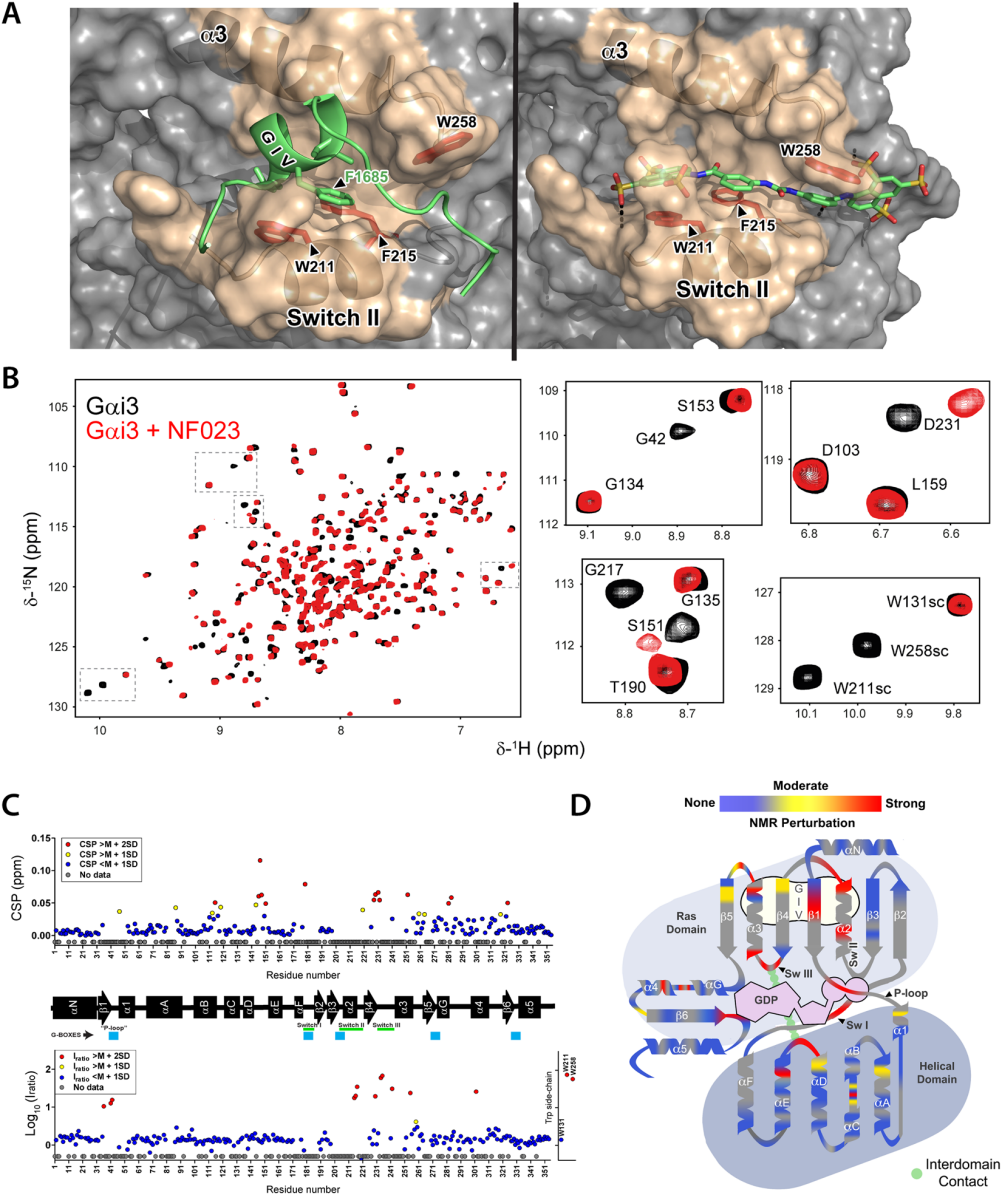
Solution NMR reveals structural perturbations in Gαi3 upon NF023 binding at single-residue resolution. (**A**) Comparison of GIV (right) and NF023 (left) binding poses on Gαi3, as seen in the virtual docking. (**B**) Overlay of ^1^H- ^15^N TROSY spectra of Gαi3 in the presence or absence of NF023. *Left*, ^1^H- ^15^N TROSY spectra of isotopically labeled [^2^H, ^15^N] Gαi3 in the absence (black) or presence of a 1:1 molar ratio of NF023 (red). *Right*, selected regions from the overlaid spectra depicting representative perturbations in Gαi3 signals induced by NF023 binding. Some signals exhibit large chemical shift changes upon NF023 binding, whereas some other signals exhibit dramatic signal intensity reductions. (**C**) Quantification of NF023-induced perturbations of the NMR signals of Gαi3. Chemical shift perturbations (CSP, top graph) or peak intensity changes (I_ratio_ = I_bound_/I_free_, bottom graph) of the backbone amide signals of the TROSY NMR spectra in panel B. Yellow and red circles indicate residues undergoing perturbations larger than the median (M) plus one or two SD, respectively. Blue circles indicate residues with perturbations below the median (M) plus SD and grey circles indicate Gαi3 residues for which no information was available. The horizontal black bar between the two graphs depicts the secondary structure elements of Gαi3 and is annotated with the position of the 3 switch regions (green) that undergo conformational changes upon GTP binding and the 5 conserved G-box sequences (blue) that mediate nucleotide binding. (**D**) Schematic representation of NF023-induced NMR perturbations on Gαi3. Gαi3 secondary structure elements were arranged to mimic their orientation relative to the nucleotide in the three-dimensional structure. Color coding is the same as in panel C and corresponds to a composite of CSP and I_ratio_ perturbations. For residues with changes in both CSP and I_ratio_ measurements, the largest of the two was taken. GDP is shown in purple and the interdomain interaction between the Switch III of the Ras-like domain and the αD-αE loop of the all-helical domain is shown in green. The predicted GIV binding region is shown in light beige.

**Figure 8:**
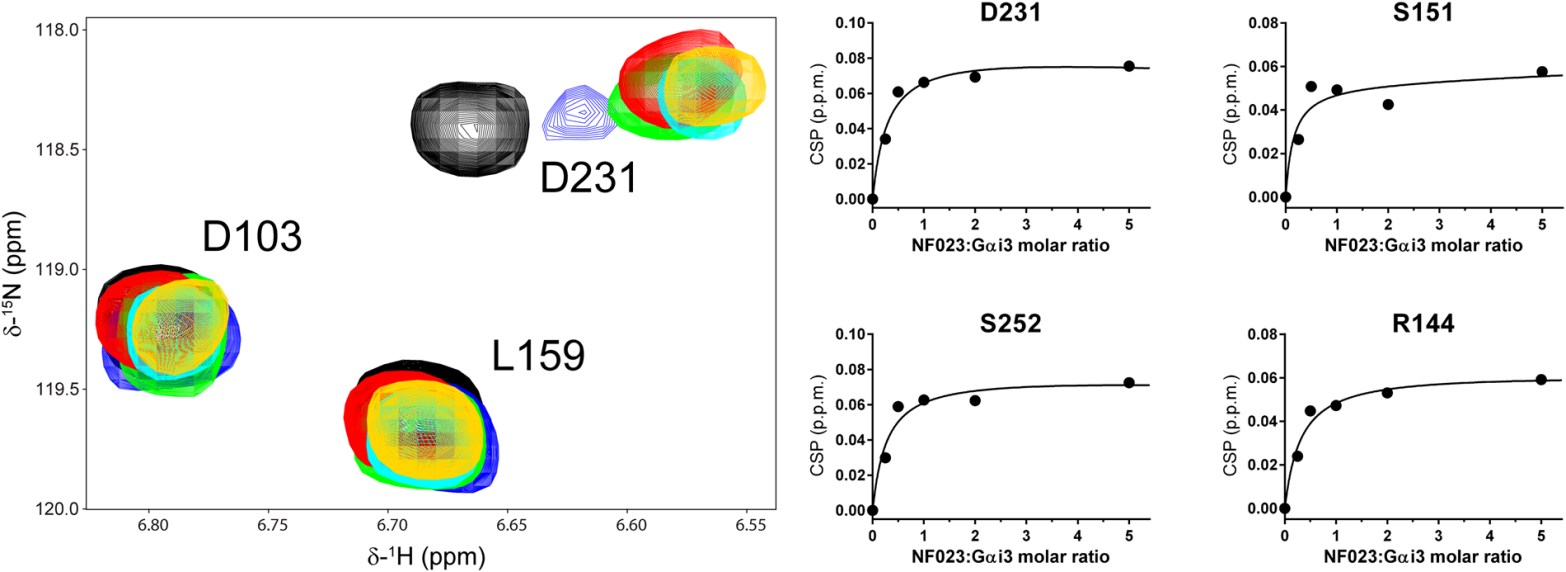
Overlay of ^1^H- ^15^N- TROSY spectra of perdeuterated Gαi3 after addition of increasing amounts of NF023. *Left*, The selected region corresponds to one of the panels in Fig. 7B and shows the shift of the D231 signal. The contour levels are the same in all spectra. The colors correspond to the molar Gαi3:NF023 ratios 1:0 (black), 1:0.25 (blue). 1:0.5 (green), 1:1 (red), 1:2 (cyan) and 1:5 (orange). *Right*, Plot of the measured CSP values of selected Gαi3 residues (circles) and fitting to a single-site binding model (lines). The experimental error was ± 0.008 ppm.

**Figure 9:**
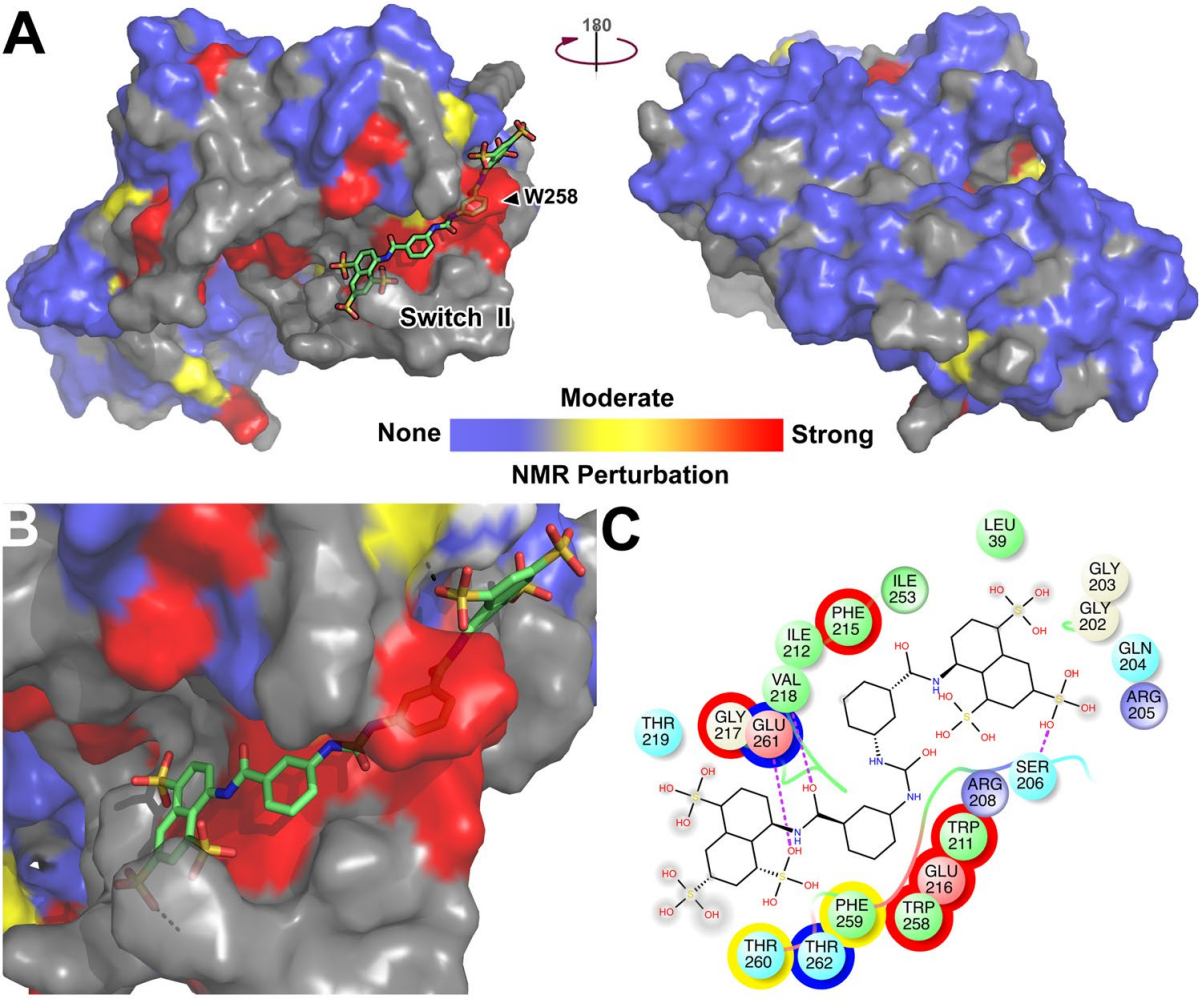
Overlay of NF023-induced NMR perturbations on a structural model of Gαi3. (**A,B**) A model of Gαi3 in the GIV-bound conformation (Fig. 1) was colored for NMR perturbations as in Fig. 7 in the presence of virtually docked NF023 (balls and sticks). A front and back surface view of Gαi3 is shown in (**A**) and an enlarged view of the predicted NF023 binding site in (**B**). **(C**) Two-dimensional diagram of NF023 and its predicted binding contacts. Spheres represent all Gαi3 residues located at <4 Å from NF023. The color fill of the spheres indicates the chemical properties of the residues (green = hydrophobic, blue = positively charged, red = negatively charged, cyan = polar, white = neutral). The color of the outer circles indicates the NMR perturbations as in Fig. 7 (no outline = no NMR data available). The diameter of the gray shadows in NF023 indicates solvent exposure of chemical groups.

Next, we used solution NMR spectroscopy to validate the binding mode of the inhibitors. NMR signals of specific amino acids are very sensitive to changes in their chemical environment and therefore inform on the site of interaction with a small molecule as well as on indirect structural rearrangements associated with its binding. Initial analyses indicated that ATA aggregates in aqueous solution and may yield experimental artifacts under the conditions used for NMR (i.e., high concentrations of compound and protein). For this reason we focused our efforts on the characterization of NF023 binding, which is predicted to extensively overlap with the GIV binding site by docking onto the SwII/α3 cleft and extending into the groove under the W258 side chain (Fig. 7A). For this purpose we recorded NMR spectra on Gαi3 uniformly enriched in ^2^H and ^15^N isotopes. The backbone amide signals for 60% of Gαi3 residues, as well as the side chain signals for its three tryptophans (W131, W211, W258), could be assigned in the ^1^H- ^15^N TROSY spectrum by reference to the published assignment of GDP-bound Gαi3^54^. The assigned signals spanned across the different domains and secondary structure elements of the G protein. ^1^H- ^15^N TROSY spectra were acquired for Gαi3 in the presence of increasing concentrations of NF023, allowing the transfer of the assignment to the NF023-bound form. Overlay of the TROSY spectra of Gαi3 free and in the presence of equimolar amounts of NF023 revealed significant Chemical Shift Perturbations (CSP) in only a fraction (<12%) of the assigned peaks (Fig. 7B,C). Titration with increasing molar ratios of NF023 (0.25 to 5 relative to Gαi3) revealed that the extent of the CSP reached near saturation values at equimolar Gαi3:NF023 ratios (Fig. 8). We also observed specific changes in the intensity (I) of some signals and analyzed the I_ratio_ = I_free_/I_bound_ to identify additional specific perturbations caused by NF023. As for CSP, we found perturbations of the I_ratio_ only in a small subset (~6%) of the assigned peaks (Fig. 7B,C). To facilitate the interpretation of these results, we mapped the NMR signal perturbations onto a 2D diagram of Gαi3 (Fig. 7D) and a 3D structural model of Gαi3 (Fig. 9). A region of Gαi3 showing marked perturbations in the presence of NF023 mapped to the SwII, α3 helix and α3/β5 loop (Fig. 7D), which were in good agreement with the predicted binding pose of NF023 on the 3D structural model (Fig. 8). This binding mode of NF023 largely overlaps with the binding area for the GBA motif of GIV predicted by computational modeling and previously validated by NMR mapping and biochemical assays^46^, thereby explaining the observed inhibition of binding. In addition to this region matching the Gαi3-GIV interface, another region showed perturbations in the presence of NF023. This region includes structural elements within or near the nucleotide binding site (Figs 7D and 9). We interpret these perturbations as secondary structural rearrangements, rather than direct NF023 contact sites.

### Biochemical validation of NF023 binding site on Gαi3

**Figure 10:**
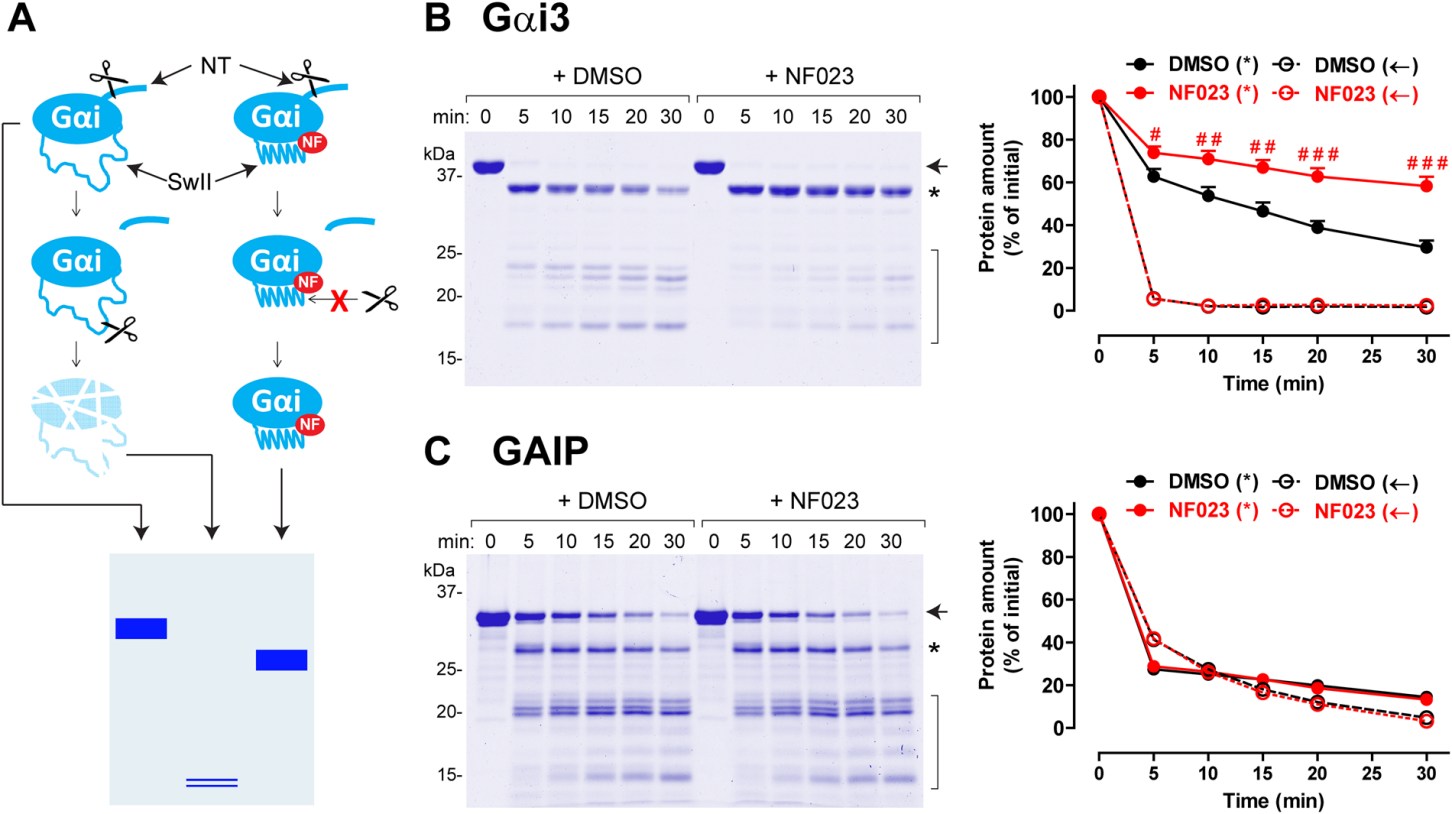
NF023 protects Gαi3 from trypsin-mediated proteolysis. (**A**) Schematic of the principle for the limited proteolysis assays. Trypsin rapidly cleaves the N-terminus of Gαi3 but digestion of the remainder of the protein depends on the conformation/protease accessibility of the SwII region. When the SwII is disordered (*left*), Gαi3 is readily digested into small fragments. When the SwII adopts an ordered conformation (*right*), it becomes protected from the action of trypsin. NF = NF023. (**B**) NF023 delays Gαi3 digestion by trypsin. His-Gαi3 was incubated with trypsin at 4 °C in the presence or absence of NF023 (25 µM) for the indicated times. The resulting products were separated by SDS-PAGE and stained with Coomassie Blue. ***Left***, one gel representative of four experiments. Arrow = full-length Gαi3; asterisk = N-terminally cleaved Gαi3; bracket = low molecular weight proteolytic fragments of Gαi3. ***Right***, quantification of limited proteolysis data shown on the right presented as mean ± S.E. of 4 independent experiments (^#^P < 0.05, ^##^P < 0.01, ^###^P < 0.001 compared to DMSO). Black = DMSO, Red = NF023. Solid lines correspond to the N-terminally cleaved Gαi3 (asterisk in the gel shown on the left) and dashed lines to full-length Gαi3 (arrows in the gel shown on the left). **(C)** NF023 does not affect GAIP digestion by trypsin. His-GAIP was incubated with trypsin at 4 °C in the presence or absence of NF023 (25 µM) for the indicated times. The resulting products were separated by SDS-PAGE and stained with Coomassie Blue. ***Left***, one gel representative of three experiments. Arrow = full-length GAIP, asterisk = larger proteolytic fragment of GAIP, Bracket = smaller proteolytic fragments of GAIP. ***Right***, quantification of limited proteolysis data shown on the right presented as mean ± S.E. of 3 independent experiments. Black = DMSO, Red = NF023. Imagens of the full gels presented in this figure are shown in Supplementary Information.

The results described above indicate that NF023 binds to the GIV binding region on Gαi3. One limitation of the analysis described above is the lack of NMR signal assignments for a large fraction of the residues in the SwII, an element of this binding region. To further validate the NMR findings and support our interpretation, we used a complementary biochemical approach based on the analysis of limited proteolysis experiments with Gαi3. The proteolytic digestion of Gαi by trypsin is well characterized^22^. The earliest proteolytic cleavage by trypsin results in the loss of the ~25 N-terminal residues of Gαi proteins. The next proteolytic cleavage occurs in the SwII region and it is a critical event that determines the fate of subsequent proteolytic events. If the cut in the SwII occurs, trypsin efficiently digests the reminder of Gαi into small molecular weight fragments, whereas if it does not occur, the protein remains resistant to trypsin digestion. The SwII is a structurally dynamic region and Gαi sensitivity to trypsin digestion can be used to probe for its different conformational states. For example, the SwII is disordered in GDP-bound Gαi^47^ whereas it adopts an ordered helical conformation in GTP-bound Gαi^55^, which make Gαi-GTP resistant to trypsin under conditions in which Gαi-GDP is readily digested (Fig. 10A). We reasoned that binding of NF023 to GDP-bound Gαi3 would result in increased resistance to trypsin proteolysis because our structural model and computational docking predict that this compound would bind and presumably stabilize a conformation of Gαi3-GDP in which the SwII adopts a defined (possibly helical) conformation. To be able to monitor the course of the rapid proteolytic digestion of Gαi3-GDP, we performed the experiments at reduced temperatures (in ice). As expected, we found that the first proteolytic cleavage that removes the N-terminus of Gαi3 occurred very rapidly (completed in <5 min) whereas the subsequent digestions dependent on the cut of SwII took longer times (T_1/2_ ~15–20 min) in control reactions (Fig. 10B). In the presence of NF023, the N-terminus was still rapidly cleaved whereas subsequent proteolysis was significantly diminished (Fig. 10B). As judged from the rate of disappearance of the N-terminally cleaved fragment, NF023 decreased >2-fold the ability of trypsin to cut in the SwII (Fig. 10B). This effect was not due to non-specific blockade of the enzymatic activity of trypsin by NF023 because the same concentration of compound did not affect the ability of trypsin to digest purified His-GAIP, another globular protein structurally unrelated to Gαi3 (Fig. 10C). Taken together, our computational predictions, NMR data and biochemical results strongly support the conclusion that NF023 works as an inhibitor of GIV binding to Gαi3 by directly binding and blocking the protein-protein interface.

### NF023 specifically inhibits the Gαi3-GIV interaction without disrupting Gαi3-Gβγ binding

**Figure 11:**
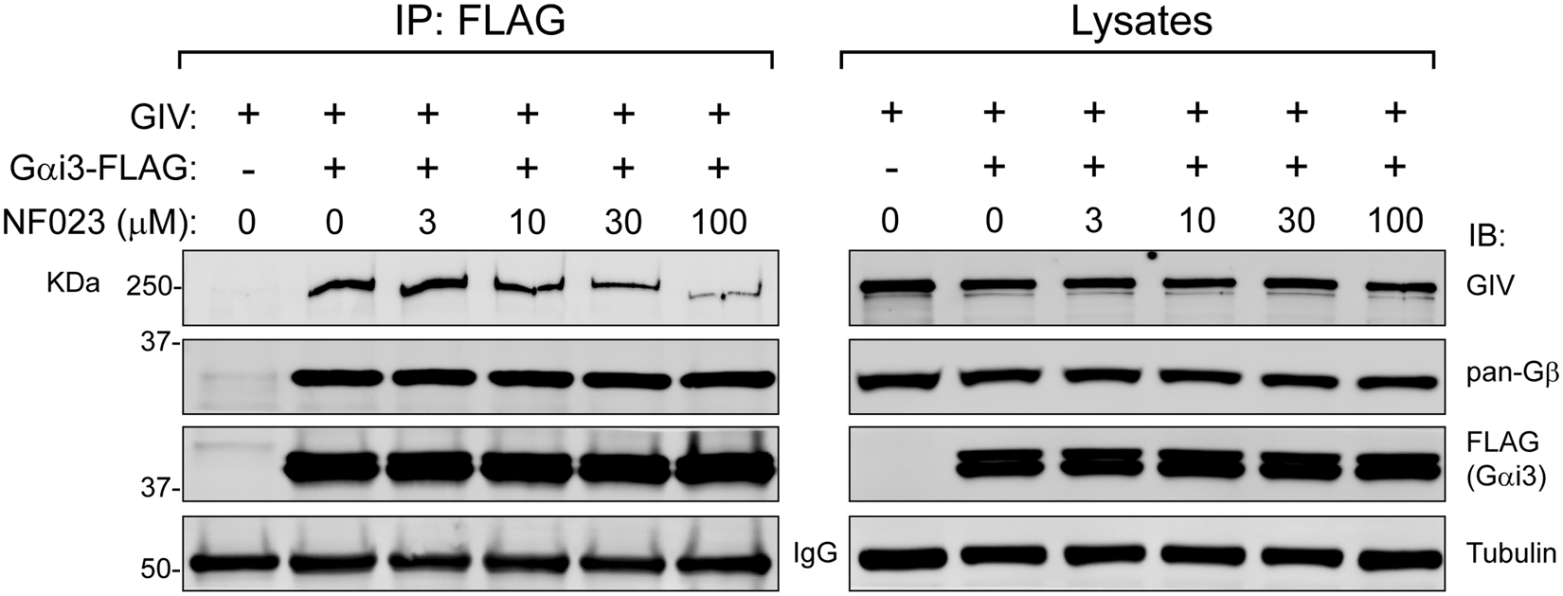
NF023 inhibits GIV but not Gβγ co-immunoprecipitation with Gαi3. Lysates of HEK293T cells transfected with Gαi3-FLAG and full-length GIV as indicated were subjected to immunoprecipitation (IP) with anti-FLAG antibodies in the presence of increasing amounts of NF023 (3–100 µM). Immunoblots of the immunoprecipitated proteins are shown on the left panels and immunoblots of equal lysate aliquots on the right panels. One experiment representative of three is shown. Imagens of the full membrane strips probed with antibody are shown in Supplementary Information.

GIV and NF023 binding sites on Gαi3 partially overlap with a major contact site for Gβγ, which is the main binding partner of GDP-bound Gαi in cells. More specifically, Gβγ makes contact with the SwII of Gαi^56^. This raises a general concern about the specificity for any chemical probe that interferes with the Gαi-GIV interaction because it might also disrupt Gαi binding to Gβγ and lead to undesired off-target effects. Freissmuth and colleagues concluded that NF023 did not disrupt Gα-Gβγ binding based on results from biochemical assays and experiments in semi-permeabilized cells that indirectly reflect heterotrimer association/dissociation^52^. We tested the effect of NF023 on Gαi3-Gβγ by direct protein-protein binding assays and compared it to the effect on Gαi3-GIV under identical conditions. For this purpose, we determined how much GIV (full-length) and Gβγ co-immunoprecipitated with Gαi3 when lysates of cells expressing the indicated proteins were supplemented with increasing concentrations of NF023. We found that binding of full-length GIV to Gαi3 was diminished by NF023 in a dose-dependent manner whereas Gβγ binding was unaffected in the same experimental samples (Fig. 11). These results demonstrate that a small molecule like NF023 can specifically disrupt the Gαi3-GIV interaction without disrupting Gαi3-Gβγ binding.

## Discusion

The main finding of this work is that the Gαi-GIV interface can be inhibited by small molecules, i.e., it is a druggable target. This is important because of the implications of this interaction in cancer cell biology and because PPIs are challenging targets whose druggability must be validated in a case-by-case basis. We not only provide the proof of principle for the druggability of the Gαi-GIV interface but also a pipeline of assays for the identification and filtering of small molecule inhibitors in a high-throughput format. Despite the lack of an atomic resolution structure of the Gαi-GIV complex, our data indicates that structural insights gained from NMR and modeling are reliable for predicting the binding of small molecules in the GIV-docking region of Gαi. Taken together, our results provide the framework for the discovery, validation and development of small molecule inhibitors for the Gαi-GIV PPI.

The finding that NF023 can disrupt GIV binding to Gαi3 without simultaneously affecting Gαi3-Gβγ supports that Gαi-GIV can be specifically targeted. Previous evidence^22^ indicated that this might be the case because mutagenesis of Gαi3 can disrupt GIV binding without affecting binding to GPCRs, Gβγ or other regulators like RGS and GoLoco proteins. Based on current knowledge, the GIV side of the Gαi-GIV interface is hardly druggable because it consists of a short sequence in a disordered region of the protein^46^, with no pocket to accommodate a small molecule. Thus, small molecule inhibitors of the Gαi-GIV interface must bind to the G protein, which in turn could interfere with the binding of other proteins. However, specificity is a concern for some but not all Gα-binding partners. For example, the binding site for GPCRs does not overlap with the GIV binding site of Gα^46^, suggesting that blocking GIV binding by small molecules might not directly affect the GPCR-G protein binding. Similarly, although effectors and GIV bind to a region of Gα composed of the same structural elements (i.e., SwII and α3)^57, 58^, they do so when they are in very different conformations. GIV binds to Gαi exclusively in its GDP-bound conformation^30^ whereas effectors bind to the GTP-bound conformation^57, 58^. Because the SwII/α3 region is structurally different between GDP- and GTP-bound conformations^47, 55^, it is reasonable to imagine that a small molecule could discriminate between the two of them. On the other hand, Gβγ binds to Gαi in its GDP-bound conformation and its binding site partially overlaps with that of GIV^30, 56^. In fact, GIV can compete with Gβγ for binding to Gαi^30^. Based on this, it might seem contradictory that NF023 does not disrupt Gβγ binding under the same conditions in which it disrupts GIV binding. The reason for this apparent inconsistency might be that the Gβγ binding surface on Gαi is very large and it overlaps only partially with the GIV binding surface. Thus, while GIV might be large enough to sterically displace Gβγ from Gα, a small molecule might not. A similar precedent exists for Gβγ inhibitors— while peptide-based inhibitors disrupt Gβγ binding to all its effector proteins, small molecules targeted to the same region of Gβγ only disrupt a subset of them^8, 9^.

Because the intention of this study was to establish the duggability of the Gαi-GIV PPI, we focused our efforts on testing a well characterized library of bioactive molecules, i.e., LOPAC. By definition, all these compounds are not specific for Gαi-GIV. NF023 and suramin have been shown to inhibit not only Gα subunits but also P2X receptors, and they are not cell permeable^52^, which precludes their use for an intracellular target like Gαi-GIV. ATA, the other validated hit, also has additional targets, like topoisomerase II. However, the LOPAC represents a curated collection of diverse molecules with drug-like properties that has been extensively used as a standard for pilot validation of targets and assays. The low number of primary hits and high rate of validation indicate that our target and assays do not yield promiscuous hits. Moreover, the structural characterization of NF023 binding supports that the Gαi-GIV interface is a defined and tractable target for which the mode of inhibition is predictable. Thus, our results using the LOPAC library support that screening of larger or PPI-focused libraries of compounds is not only feasible but also likely to yield inhibitors with the desired properties. In addition to identifying small molecules that selectively block the Gαi-GIV interaction and that can act intracellularly, assessing their possible impact on normal physiological functions linked to GIV and/or Gαi will be crucial to further pursue this target in the context of cancer experimental therapeutics. Although the role of the GIV-Gαi signaling axis in normal physiology has not been well characterized, the upregulation of GIV in metastatic cancers holds the promise of providing a therapeutic window for intervention.

## Methods

### Computational structure model of the Gαi3-GIV interface

A structural model of GIV’s GBA motif (residues 1678–1696) bound to human Gαi3 was constructed as previously described^46^. Briefly, the crystal structure of human Gαi1 in complex with the GBA-like synthetic GEF peptide KB-752 (PDB: 1Y3A) was used as the template to build homology models of Gαi3 and GIV 1678–1688. Fast Fourier transform protein-protein docking simulations were performed with ICM version 3.8–3 (Molsoft LLC., San Diego, CA). The docked GIV model was further extended at the C-terminus with Monte-Carlo based *ab initio* folding within the Gαi3 context to predict residue contacts with the full Gαi3 binding pocket not represented by the KB-752 crystal structure template. The extended GIV sequence was re-docked as above and the model was further refined with a Fragment-Guided Molecular Dynamics (FG-MD) simulation^59^. FoldX version 3.0 was used to identify and repair high-energy side-chain conformations^60^. icmPocketFinder was used to predict small molecule-accessible druggable sites on the patch of the Gαi3 surface where GIV docks on. Protein structure image displays were prepared using either ICM (Molsoft) or PyMol (DeLano).

### Protein purification

Rat or human His-Gαi3, rat GST-Gαi3, human His-GAIP, human His-GIV-CT (1660–1870) and GST-GIV (1671–1755) were expressed in BL21(DE3) *E. coli* transformed with previously described plasmids^22, 51, 54^ by overnight induction at 23 °C with 1 mM isopropyl-β-D-1-thio-galactopyranoside (IPTG). Protein purification was carried out following previously described protocols^22, 30^. Briefly, pelleted bacteria from 1 L of culture were resuspended in 25 ml of buffer [50 mM NaH_2_PO_4_, pH 7.4, 300 mM NaCl, 10 mM imidazole, 1% (v:v) Triton X-100 supplemented with protease inhibitor cocktail (Leupeptin 1 µM, Pepstatin 2.5 µM, Aprotinin 0.2 µM, PMSF 1 mM)]. For Gαi3, this buffer was supplemented with 25 µM GDP and 5 mM MgCl_2_. After sonication (four cycles, with pulses lasting 20 s/cycle, and with 1 min interval between cycles to prevent heating), lysates were centrifuged at 12,000 × g for 20 min at 4 °C. Solubilized proteins were affinity purified on HisPur Cobalt or Glutathione Agarose resins (Pierce) and eluted with lysis buffer supplemented with 250 mM imidazole or 50 mM Tris-HCl,pH 8, 100 mM NaCl, 30 mM reduced glutathione, respectively. GIV proteins were dialyzed overnight at 4 °C against PBS. For Gαi3 proteins, the buffer was exchanged for 20 mM Tris-HCl, pH 7.4, 20 mM NaCl, 1 mM MgCl_2_, 1 mM DTT, 10 μM GDP, 5% (v/v) glycerol using a HiTrap Desalting column (GE Healthcare). Protein samples were aliquoted stored at −80 °C.

Untagged rat Gαi3 (expressed from a pET28b plasmid containing a thrombin cleavage site) was prepared by incubating rat His-Gαi3 with agarose-immobilized thrombin (Sigma, RECOMT) at room temperature for 1 hr in buffer supplemented with 10% (v:v) glycerol. After centrifugation, the supernatant was collected and incubated with HisPur Cobalt beads to absorb the cleaved His-tags and uncleaved His-Gαi3. The unbound fraction was purified by gel filtration chromatography using a SuperdexS200 column equilibrated with 20 mM Tris-HCl, pH 7.4, 20 mM NaCl, 1 mM MgCl_2_, 1 mM DTT, 10 μM GDP, 5% (v/v) glycerol.

Purification of human Gαi3 for NMR experiments was carried out as previously described^46, 54^. Briefly, protein expression in BL21 Rosetta cell was induced with 1 mM IPTG for 16 h at 23 °C in medium with deuterated water containing ^15^N-NH_4_Cl, ^2^H- ^13^C-glucose and ^2^H- ^15^N Celtone base powder. Purification was carried out by affinity chromatography using a Ni^2+^-loaded resin, cleavage of the N-terminal His-tag after elution, removal of the protease and uncleaved protein, and gel filtration chromatography on a Superdex S200 column equilibrated with 10 mM HEPES, 150 mM NaCl, 1 mM DTT, 20 µM GDP at pH 7.0.

### Peptide synthesis

Peptides corresponding to GIV fragments 1671–1689, aa1671–1696, 1671–1701, 1671–1705 (KTGSPGSEVVTLQQFLEES ^1689^NKLTSVQ ^1696^IKSSS ^1701^QENL ^1705^) or GIV 1671–1705 F1685A (KTGSPGSEVVTLQQALEESNKLTSVQIKSSSQENL) were synthesized using the *in situ* neutralization protocol for Boc-Solid Phase Peptide Synthesis (Boc-SPPS)^61^ on a *p*-methylbenzhydrylamine (MBHA) resin (Novabiochem, 0.67 mmol/g, 100–200 mesh). Following chain elongation, the peptide was cleaved from the resin using a solution of hydrofluoric acid containing a 5% of anisole for 1 h at 0 °C. Next, the solution was removed under vacuum and the resulting residue crushed out with Et_2_O and filtered. The collected solid was redisolved in a 50% CH_3_CN/H_2_O solution containing 0.1% of trifluoroacetic acid (TFA), frozen down and lyophilized. Crude peptides were purified by reverse phase-HPLC in a Waters X-Bridge C18 (19 × 100 mm) column at a flow of 20 mL/min using H_2_0 (0.1% TFA) and CH_3_CN (0.1%TFA) as eluents. The identity and final purity (>97%) of the peptide was determined by analytical RP-HPLC and mass spectrometry (ESI-TOF). Fluorescently-labeled peptide were synthesized using the same protocol except that following chain elongation 5,6-carboxyfluorescein was activated with HATU and coupled to the resin-bound peptide at 65 °C for 1 h to yield the fluorescein-labeled peptides.

### *In Vitro* Protein Binding Assays with GST-fused Proteins

GST pulldown assays were carried out as previously described^22^ with minor modifications. Ten μg of GST or GST-GIV were immobilized on glutathione agarose beads for 90 min at room temperature in PBS. Beads were washed twice with PBS, resuspended in 250 µl (final concentration ~1.25 µM) of binding buffer (50 mM Tris-HCl, pH 7.4, 100 mM NaCl, 0.4% (v:v) NP-40, 10 mM MgCl_2_, 5 mM EDTA, 2 mM DTT, 30 µM GDP) and incubated 4 h at 4 °C with constant rotation in the presence of rat His-Gαi3 (final concentrations 0.1–5 µM). Beads were washed four times with 1 ml of wash buffer (4.3 mM Na_2_HPO_4_, 1.4 mM KH_2_PO_4_, pH 7.4, 137 mM NaCl, 2.7 mM KCl, 0.1% (v/v) Tween-20, 10 mM MgCl_2_, 5 mM EDTA, 1 mM DTT and 30 µM GDP) and resin-bound proteins eluted with Laemmli sample buffer by incubation at 37 °C for 10 min. Proteins were separated by SDS-PAGE and stained with Coomassie blue. Experiments testing the effect of compounds on His-Gαi3 binding to GST-GIV were carried out the same way with 0.2 µM His-Gαi3 in the presence of 100 µM of each compound (or an equivalent volume of DMSO). Proteins were transferred to PVDF membranes followed by sequential incubation with primary (mouse anti-his, Sigma H1029, 1:2,500) and secondary antibodies (goat anti-mouse IRDye 800 F(ab′)2, Li-Cor Biosciences, 1:10,000). Images were acquired in an Odyssey infrared scanner (Li-Cor), processed using the Image J software (NIH) and assembled for presentation using Photoshop and Illustrator software (Adobe).

### Fluorescence polarization assay and high-throughput screen

Fluorescence polarization measurements were carried out in 384-well plates (Black OptiPlate-384F, Perkin Elmer). Fluorescently-labeled peptides (0.025 µM) were mixed with rat His-Gαi3 (at concentrations indicated in the figures and figure legends) at room temperature for 10 min in a final volume of 20 µl of binding buffer (50 mM Tris-HCl, pH 7.4, 100 mM NaCl, 0.4% (v:v) NP-40, 10 mM MgCl_2_, 5 mM EDTA, 2 mM DTT, 30 µM GDP). In some experiments, unlabeled peptides, purified proteins and compounds were present in the binding reaction during the incubation at room temperature and subsequent steps. For the AlF_4_^−^ conditions, the buffer was supplemented with 30 µM AlCl_3_ and 10 mM NaF. Fluorescence polarization (Ex 485 ± 10 nm/Em 528 ± 10 nm) was measured every 2 min for 30 min at room temperature in a Biotek H1 synergy plate reader to ensure that the signals were stable in time. Fluorescence polarization at different times was averaged and fitted to a one-site binding model equation to determine the equilibrium dissociation constant (K_d_) using Prism (GraphPad). For conditions not reaching binding saturation (e.g., in the presence of AlF_4_^−^), the maximal binding of His-Gαi3 measured in the same experimental set was considered maximum binding for curve fitting. For small molecule dose-inhibition curves, compounds were diluted in the same final concentration of DMSO and data was normalized to maximum binding (100%, in the absence of compound) and fitted to a one-site competitive binding model inhibition curve to calculate the IC_50_ values using Prism (GraphPad).

For high-throughput screening of the LOPAC library (Sigma, LO1280), compounds (10 µM) were transferred to 384-well plates using a liquid handler (Tecan Fredom Evo), and then rat His-Gαi3 (1 µM) and fluorescently-labeled GIV residues 1671–1701 (0.025 µM) were sequentially added using a microplate dispenser (Biotek Multiflo) in a final volume of 20 µl. The final concentration of DMSO was 1% (v:v) [the assay tolerates >5%, *not shown*]. Plates were read 15–45 min after mixing [the signal is stable for hours, *not shown*] in a Tecan Infinite M1000 Pro. Each compound was tested in triplicate and each plate contained 32 wells of positive (+AlF_4_^−^) and negative (no compound) controls (shown in Fig. 3B). The positive and negative control data points were used to calculate the Z’ (a.k.a. Z-factor) using the formula Z’ = 1−[3*(δ_positive +_ δ_negative)_/│µ_positive -_ µ_negative_│], where δ is the standard deviation and µ is the mean.

### Virtual ligand screening

The computational model of Gαi3 in the GIV-bound conformation was prepared for virtual docking using ICM by removal of GIV and water molecules followed by optimization of the isomeric/tautomeric state and positioning of histidine, glutamine, asparagine, cysteine and proline residues as well as polar hydrogens. All simulations occurred in a continuous dielectric solvent model (i.e. no explicit water). Rigid receptor small molecule docking simulations were performed via the internal coordinate mechanics^62^ in continuous dielectric solvent. Energy potentials and force field parameters are derived from the modified Merck Molecular Force Field (MMFF) for small molecules^63^ and the Empirical Conformation Energy Program for Peptides (ECEPP/3) for proteins^64^. Grid potentials were built implicitly with anisotropic van der Waals (vdW), electrostatic, hydrogen bonding and hydrophobic energy terms. Maximum vdW repulsion for receptor map was set to 4.0. Docking proceeds via a rigid receptor biased probability Monte-Carlo minimization procedure. Run termination for each ligand is adaptive based on size and flexibility and was set to sampling thoroughness factor 1. Scoring components were: internal force field energy (ligand), conformational entropy loss (ligand), receptor-ligand hydrogen bonding, solvation energy change (global) and hydrophobic energy (global). The LOPAC1280 virtual chemical library (Sigma) was used for virtual screening. No exclusions to the library were made. All compounds were scored (i.e. no hit threshold).

### AlphaScreen® protein-protein binding assay

Nickel-chelate AlphaScreen donor beads (PerkinElmer) and glutathione AlphaLISA acceptor beads (PerkinElmer) were combined with GST-Gαi3 in binding buffer (50 mM Tris-HCl, pH 7.4, 100 mM NaCl, 0.4% (v:v) NP-40, 5 mM MgCl_2_, 2 mM DTT, 30 µM GDP) and incubated 30 min in the dark at room temperature in the presence or absence of compounds, DMSO or AlF_4_^−^ (30 µM AlCl_3_, 10 mM NaF) or untagged Gαi3. His-GIV-CT (1660–1870) was added at the same concentration as GST-Gαi3 and incubated in the dark at room temperature for 2.5 h before reading. The final concentration of DMSO was 1% (v:v) [the assay tolerates at least 5%, *not shown*]. All steps were carried out in 384-well plates (White ProxyPlate-384, Perkin Elmer) and the final volume was 20 µl. AlphaScreen® chemiluminescent signals were measured in an EnVision plate reader (Perkin Elmer) by exciting at 680 nm and collecting 615 nm light. Z’ was calculated as described in “*Fluorescence polarization assay and high-throughput screen*”.

### NMR Spectroscopy

All NMR data were measured on a Bruker Avance III 800 MHz (18.8 T) spectrometer equipped with a cryogenically cooled triple resonance z-gradient probe, processed with Topspin, and analyzed with Sparky. Proton chemical shifts were referenced to internal 2,2-dimethyl-2-silapentane-5-sulfonate (DSS, 0.00 ppm), and ^15^N chemical shifts were indirectly referenced to DSS^65^. Spectra were recorded at 30 °C on ^2^H-^15^N-Gαi3 (38 µM) in 10 mM HEPES pH 7.0 with 10 mM MgCl_2_, 300 µM GDP, 5 mM DTT, 0.01% NaN_3_ and 5% ^2^H_2_O in the presence of increasing amounts of NF023 from a stock in the same buffer plus 50% DMSO. ^1^H- ^15^N TROSY spectra of Gαi3 allowed transferring most of the assignments of the protein backbone resonances deposited in the BiomagResDataBase entry 19015^46, 54^. We observed, however, a systematic offset of −1.1 and 0.09 ppm (for ^15^N and ^1^H) in our spectra with respect to the published chemical shifts. The assignment of Gαi3 resonances in the presence of an equimolar NF023 was achieved from the joint analysis of ^1^H- ^15^N TROSY spectra recorded along a titration (at Gαi3:NF023 molar ratios 1:0, 1:0.25,1:0.5, 1:1, 1:2 and 1:5; final [DMSO] = 1.1%). Most of the signal shift occurs already at ratio 1:1, indicating that the spectrum at this ratio represents the NF023 bound form of Gαi3. The chemical shift perturbations were computed as the weighted average distance between the backbone amide ^1^H and ^15^N chemical shifts in the free and bound states^66^. To compare the intensity of Gαi3 signals in different spectra, the absolute values were divided by the intensity of the C-terminal Y354, a narrow and intense signal that remained essentially unchanged upon NF023 binding. The intensity ratios (I_ratio_) for each signal were calculated by dividing the normalized intensity in the free form (I_free_) by the normalized intensity in the bound form (I_bound_).

### Limited proteolysis assay

This assay was adapted from previously described protocols^22^. Briefly, human His-Gαi3 (0.5 mg/ml) or human His-GAIP was incubated for 15 min at room temperature in the presence of 25 µM NF023 or an equivalent volume of DMSO (0.25% v:v) in in assay buffer (20 mM Na-HEPES, pH 8, 100 mM NaCl, 1 mM EDTA, 10 mM MgCl_2_, 30 µM GDP 1 mM DTT, 0.05% (w:v) C_12_E_10_). After this incubation, tubes were transferred to ice, trypsin added to the tubes (25 µg/ml or 12.5 µg/ml for His-Gαi3 or His-GAIP, respectively) and samples withdrawn at different time points (0, 5, 10, 15, 20 and 30 min). Reactions were stopped by addition of Laemmli sample buffer and boiling for 2 minutes. Proteins were separated by SDS-PAGE and stained with Coomassie blue. Images and band intensities were obtained with an Odyssey infrared scanner (Li-Cor). Images were processed using the Image J software (NIH) and assembled for presentation using Photoshop and Illustrator software (Adobe).

### Co-immunoprecipitation

This assay was performed as previously described^22, 31^. HEK293T cells were grown at 37 °C in DMEM supplemented with 10% FBS, 100 U/ml penicillin, 100 µg/ml streptomycin, 1% L-glutamine and 5% CO_2_ and transfected in 10-cm dishes with plasmids encoding for full-length GIV (7 µg, pLVX-GIV-2xMyc^40^) and FLAG-tagged Gαai3 (7 µg, p3xFLAG-CMV14-Gαi3^22^) using the calcium phosphate method. Twenty-four hours after transfection, cells from each 10-cm plate were lysed in 0.65 ml of ice-cold buffer (20 mM HEPES, pH 7.2, 5 mM Mg(CH_3_COO)_2_, 125 mM K(CH_3_COO), 0.4% Triton X-100, 1 mM DTT), vortexed and passed through a 30 G insulin syringe 5 times. After centrifugation at 14,000 × g for 10 min at 4 °C, the supernatant was collected and centrifuged again at 21,000 × g for 5 min at 4 °C. A volume of cleared lysate corresponding to cells from approximately one sixth a 10-cm dish was used for each experimental condition. NF023 (0–100 µM) was added to the lysates (final DMSO concentration was 1%, v:v) along with 2 µg of anti-FLAG mouse IgG (Sigma, F1804) and incubated 4 h at 4 °C with constant rotation. Protein G agarose beads (Thermo-Scientific) were blocked with 5% BSA for 2 hrs at room temperature, washed and added to each of the tubes containing lysates and antibodies and incubated for 90 minutes at 4 °C with rotation. Beads were then washed 3 times with wash buffer (4.3 mM Na_2_HPO_4_, 1.4 mM KH_2_PO_4_, pH 7.4, 137 mM NaCl, 2.7 mM KCl, 0.1% (v/v) Tween 20, 10 mM MgCl_2_, 5 mm EDTA, 1 mM DTT) and proteins eluted by boiling in Laemmli sample buffer for 5 min. Proteins were separated by SDS-PAGE, transferred to PVDF membranes and immunoblotted with mouse anti-FLAG, rabbit anti-GIV/Girdin (Santa Cruz Biotechnology, T-13), rabbit anti-pan-Gβ (Santa Cruz Biotechnology M-14, which reacts with all 4 canonical Gβ subunits, Gβ_1–4_) and mouse anti-tubulin (Sigma, T6074) primary antibodies followed by incubation with goat anti-rabbit and goat anti-mouse Alexa Fluor 680 (Lifetechnologies) or IRDye 800 F(ab′)2 (Licor) secondary antibodies and imaging with an Odyssey Infrared Imaging System. Images were processed using the Image J software (NIH) and assembled for presentation using Photoshop and Illustrator software (Adobe).

## Electronic supplementary material

Supplementary Information
