# Supplementary material for: The Gαi-GIV binding interface is a druggable protein-protein interaction
Source: Sci Rep. 2017 Aug 17;7:8575. doi: 10.1038/s41598-017-08829-7 (PMC5561080; doi:10.1038/s41598-017-08829-7)
Supplement: Supplementary file 1 — Supplementary Information [file 41598_2017_8829_MOESM1_ESM.pdf]

The Gαi-GIV binding interface is a druggable protein-protein interaction

Vincent DiGiacomo[1], Alain Ibáñez de Opakua[2], Maria P. Papakonstantinou[1], Lien T. Nguyen[1], Nekane Merino[2], Juan B. Blanco-Canosa[3], Francisco J. Blanco[2,4], Mikel Garcia-Marcos[1*]

[1]Department of Biochemistry, Boston University School of Medicine, Boston, USA
[2]CIC-BioGune, Derio, Spain
[3]Department of Chemistry and Molecular Pharmacology, IRB Barcelona, Spain
[4]IKERBASQUE, Basque Foundation for Science, Bilbao, Spain

SUPPLEMENTARY INFORMATION

- **Supplementary Figure 1-** GST-GIV 1671-1701 F1685A has impaired His-Gαi3 binding compared to GST-GIV 1671-1701 WT.
- **Supplementary Figure 2-** DMSO tolerance test for the interaction between GIV 1671-1701 and His-Gαi3 in FP assays.
- **Supplementary Figure 3-** NF449 inhibits the interaction between GIV 1671-1701 and His-Gαi3 in FP assays.
- **Supplementary Figure 4-** DMSO tolerance test for the interaction between His-GIV 1660-1870 and GST-Gαi3 in AlphaScreen assays.
- **Supplementary Figure 5-** Suramin is less efficient than NF023 in inhibiting the interaction between His-GIV 1660-1870 and GST-Gαi3 in AlphaScreen assays.
- **Supplementary Figure 6-** Uncropped original images of gels and/or membranes.

SUPPLEMENTARY FIGURE 1

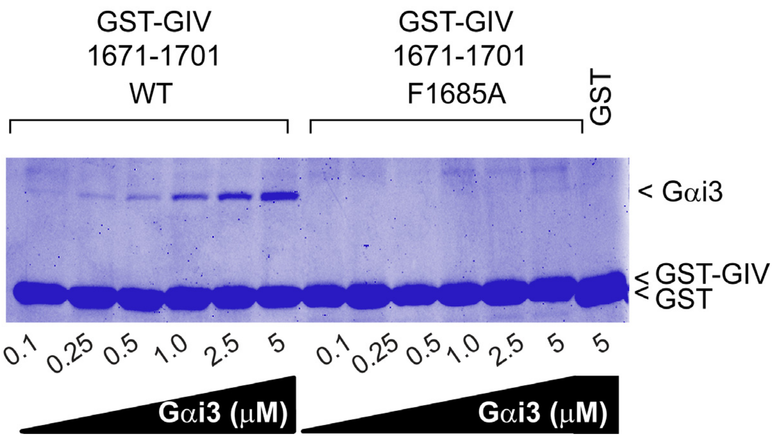

Supplementary Figure 1. GST-GIV 1671-1701 F1685A has impaired His-Gαi3 binding compared to GST-GIV 1671-1701 WT. Increasing concentrations of purified His-Gαi3 (0.1-5 μM) were incubated with GST-GIV 1671-1701 WT, GST-GIV 1671-1701 F1685A or GST and binding determined by pulldown assays as described in *Methods*. GST-GIV 1671-1689 showed diminished His-Gαi3 binding compared to the other GST-GIV proteins. One representative experiment out of two is shown.

SUPPLEMENTARY FIGURE 2

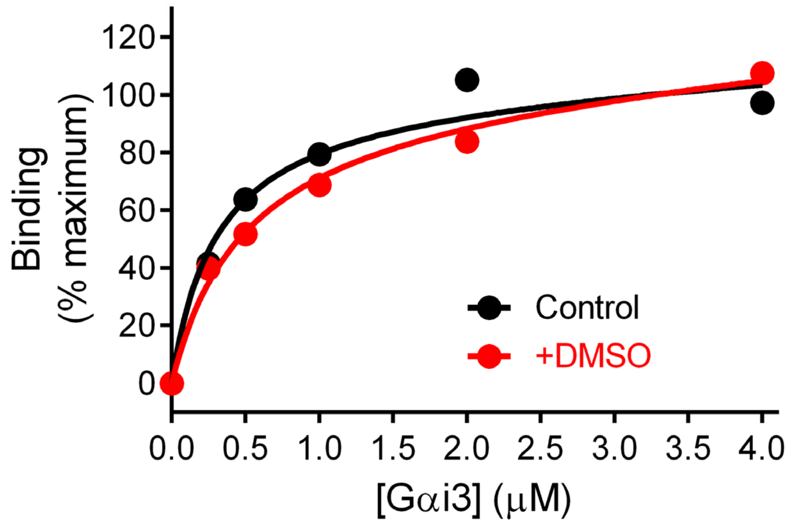

Supplementary Figure 2. DMSO tolerance test for the interaction between GIV 1671-1701 and His-Gαi3 in FP assays. Increasing concentrations of purified His-Gαi3 (0.25-4 μM) were incubated with the fluorescently-labeled peptide GIV 1671-1701 (25 nM) in the absence (black) or presence of 2.5% (v:v) of DMSO. Fluorescence polarization (FP) data were fit to a one-site binding model.

SUPPLEMENTARY FIGURE 3

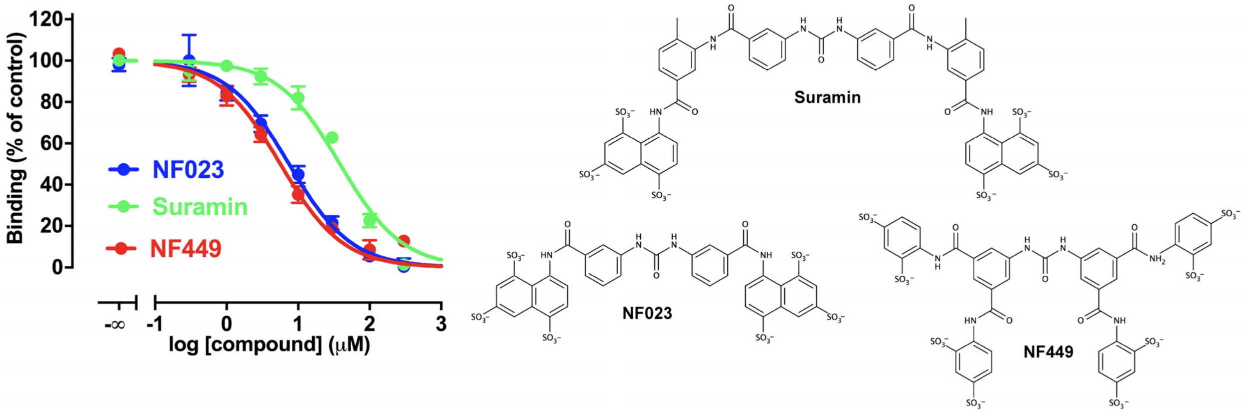

Supplementary Figure 3. NF449 inhibits the interaction between GIV 1671-1701 and His-Gαi3 in FP assays. Suramin, NF446 and NF023 were tested at different concentrations (0.3-300 μM) in FP assays. FP data was normalized relative to maximal binding in the absence of compounds and fitted to a one-site sigmoidal inhibition curve as described in *Methods*. Results are expressed as mean ± S.E. of 4 independent experiments. Chemical structures of the three compounds are shown on the left.

SUPPLEMENTARY FIGURE 4

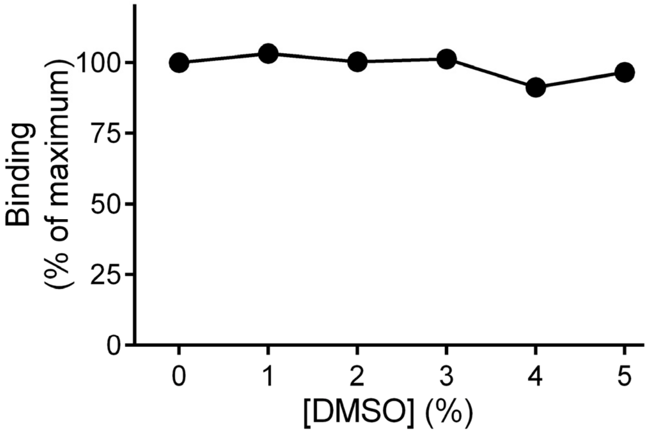

Supplementary Figure 4. DMSO tolerance test for the interaction between His-GIV 1660-1870 and GST-Gαi3 in AlphaScreen assays. 75 nM proteins (His-GIV-CT and GST-Gαi3 protein), 10 μg/mL of donor beads and 5 μg/mL of acceptor beads were incubated in the presence of the indicated concentrations of DMSO. Data points correspond to the average of three technical replicates of one representative experiment out of two with almost identical results.

SUPPLEMENTARY FIGURE 5

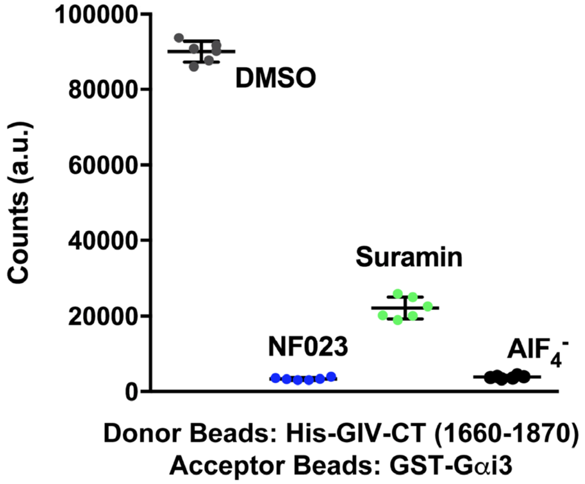

Supplementary Figure 5. Suramin is less efficient than NF023 in inhibiting the interaction between His-GIV 1660-1870 and GST-Gαi3 in AlphaScreen assays. 75 nM proteins (His-GIV-CT and GST-Gαi3 protein), 10 μg/mL of donor beads and 5 μg/mL of acceptor beads were incubated in the presence of the indicated compounds (100 μM) or an equivalent volume of DMSO. Conditions with AlF_4^- were also included as positive controls. Data points correspond to six technical replicates of one representative experiment out of two with almost identical results.

SUPPLEMENTARY FIGURE 6

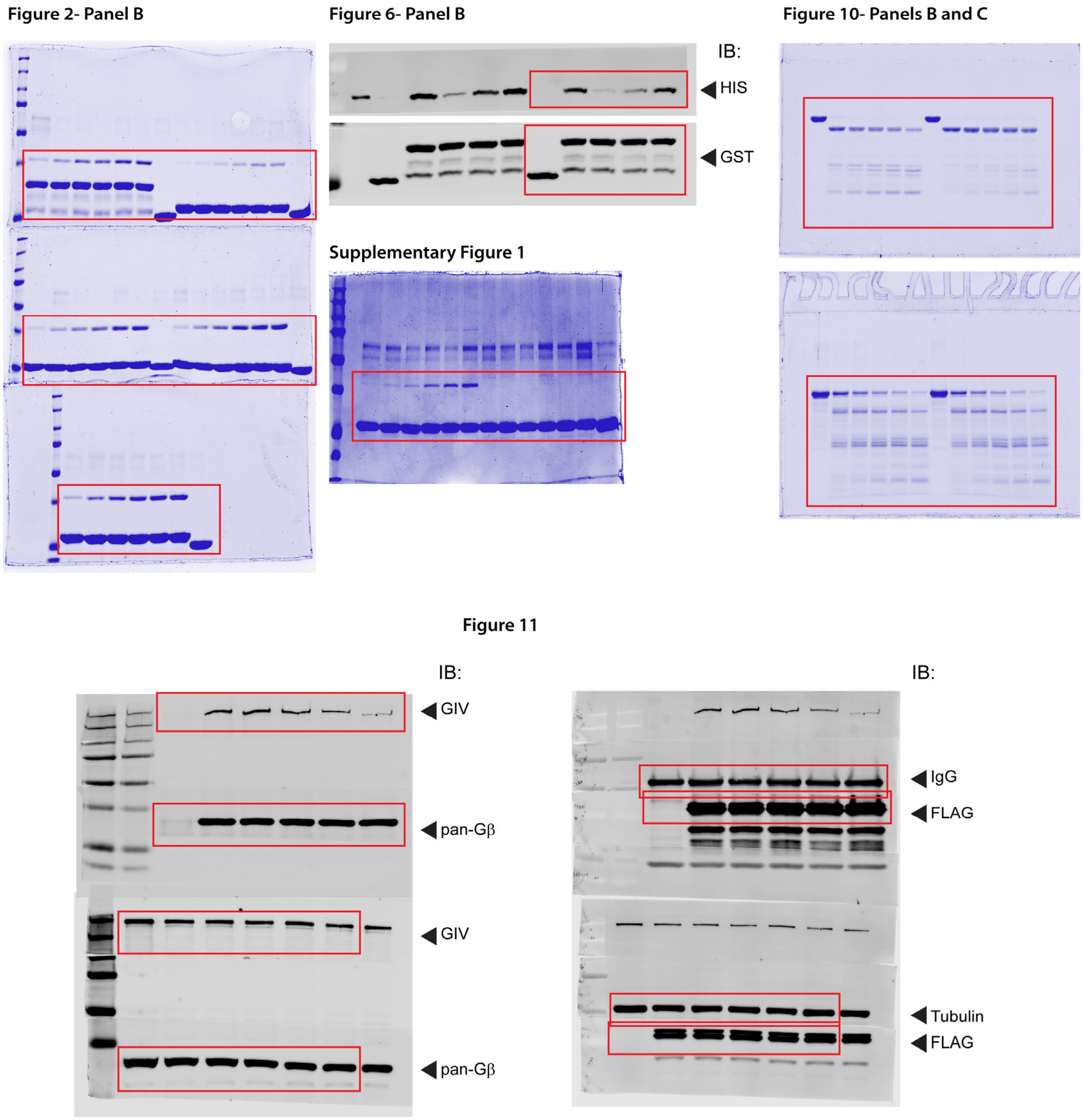

Supplementary Figure 6. Uncropped original images of gels and/or membranes. Red boxes indicate the parts of that are shown in the corresponding final immunoblot figures.
